# No causal effect of school closures in Japan on the spread of COVID-19 in spring 2020

**DOI:** 10.1038/s41591-021-01571-8

**Published:** 2021-10-27

**Authors:** Kentaro Fukumoto, Charles T. McClean, Kuninori Nakagawa

**Affiliations:** 1 Department of Political Science, Gakushuin University, Tokyo, Japan; 2 Program on U.S.-Japan Relations, Harvard University, Cambridge, MA USA; 3 Center for Japanese Studies, University of Michigan, Ann Arbor, MI USA; 4 Department of Economics, Shizuoka University, Shizuoka, Japan

**Keywords:** Government, Viral infection, Education, Respiratory tract diseases, Interdisciplinary studies

## Abstract

Among tool kits to combat the coronavirus disease 2019 (COVID-19), caused by severe acute respiratory syndrome coronavirus 2, school closures are one of the most frequent non-pharmaceutical interventions. However, school closures bring about substantial costs, such as learning loss. To date, studies have not reached a consensus about the effectiveness of these policies at mitigating community transmission, partly because they lack rigorous causal inference. Here we assess the causal effect of school closures in Japan on reducing the spread of COVID-19 in spring 2020. By matching each municipality with open schools to a municipality with closed schools that is the most similar in terms of potential confounders, we can estimate how many cases the municipality with open schools would have had if it had closed its schools. We do not find any evidence that school closures in Japan reduced the spread of COVID-19. Our null results suggest that policies on school closures should be reexamined given the potential negative consequences for children and parents.

## Abstract (web summary)

School closures in municipalities in Japan at the start of the COVID-19 pandemic showed no significant reduction in cases compared with case counts in municipalities with open schools, questioning the utility of school closures in mitigating community spread of severe acute respiratory syndrome coronavirus 2.

## Main

School closures have been widely implemented as a non-pharmaceutical intervention (NPI) to reduce the spread of COVID-19 caused by severe acute respiratory syndrome coronavirus 2 (SARS-CoV-2). By April 2020, 1 month after the World Health Organization (WHO) characterized COVID-19 as a pandemic, 173 countries had closed schools, affecting 84.3% of the world’s enrolled students^1^. Yet, school closures have broad impacts, including learning loss (as evidenced by the decrease in time spent learning^2^ and test scores^3^), future earnings loss^4^, deterioration of physical health (for example, cancellation of school meals^5^ and increase in weight^6^) as well as mental health^7,8^, maltreatment^9^ and lower maternal labor force participation^10^, including health-care workers^11^. Notably, these costs are disproportionately imposed on disadvantaged families, thereby widening social and economic inequality^2–4^. Furthermore, school closures will lead to even long-term macroeconomic damage^4,12^.

Accordingly, it is imperative to know whether the benefits of school closures outweigh these costs. Nonetheless, scholars have not reached a consensus on the degree of benefit, if any, to closing (or not reopening) schools (see a recent systematic review^13^). Some simulation^14–19^ and empirical^20–33^ studies show that school closures are effective in mitigating the spread of COVID-19. However, others fail to establish such statistically significant effects^33–49^.

We argue that one of reasons why the literature is equivocal is methodological. Simulation studies assume parameters in their models, whose values may not be correct. Most empirical works estimate parameters (including the effect of school closures) by using publicly available aggregated data, although these studies are not necessarily rigorous in terms of causal inference. A typical research design is panel regression: using a dataset that spans across countries and days, researchers regress the number of cases on a dummy variable to indicate whether a country closes its schools on a given day, where the coefficient of the dummy represents the effect of school closures. In essence, researchers estimate the effect of school closures by measuring the difference in the number of cases between days when a country closes and opens its schools. Many articles do not control for any other variables, while others include only a few control variables. Therefore, readers should be concerned about dozens of potential confounders that affect both school closures and the number of cases (for example, share of children in the population, medical preparedness and the government’s fiscal situation), which would bias estimates of the effect of school closures. Relatedly, we cannot rule out the possibility of reverse causality, namely, that governments close their schools exactly because of a high number of COVID-19 cases among their residents^42^. If this is true, naive regressions with few control variables would underestimate the effect of school closures on infection. Moreover, in essence, panel regression exploits variation in school closures across space and time, but school closures usually coincide with other NPIs (such as stay-at-home orders and prohibitions on gatherings) and/or are introduced simultaneously nationwide. Thus, it is challenging to disentangle the effect of school closures from those of other NPIs and/or from other contemporary factors such as season, economy and weather, especially when the unit of analysis is as large as a country or state^13,27,32,35^.

Here, to estimate the causal effect of school closures on reducing the spread of COVID-19, we use data from Japan, where some municipalities closed their schools, while others did not. We exploit this variation in school closures among hundreds of municipalities by utilizing matching techniques to account for dozens of confounders^50^ (Table 1).

**Table 1:** Policy summary

Background
Studies have not reached a consensus about the effectiveness of school closures on reducing the spread of COVID-19, partly because of analytical challenges. To estimate the causal effects strictly in Japan in spring 2020, we compared the number of cases between municipalities with open schools and municipalities with closed schools, both of which were selected so that they are similar in terms of dozens of features (for example, past number of cases; social, economic and political factors; and NPIs other than school closures such as stay-at-home warnings and business closures).
Main findings and limitations
On average, the numbers of cases per 100,000 residents in municipalities with closed schools were not statistically significantly smaller than those in municipalities with open schools. Because both groups are comparable except for school closure status, the difference between them should only be attributed to school closure status. Thus, we did not find any evidence that school closures significantly decreased the spread of COVID-19. It is possible that school closures lowered COVID-19 cases outside Japan and/or after spring 2020, in particular in the presence of new variants of SARS-CoV-2.
Policy implications
Our null results concerning the supposed benefits of closing schools suggest that policymakers should be cautious when considering similar policies in the future, especially given the substantial costs to the well-being of both children and parents. Our recommendation is that governments should monitor SARS-CoV-2 infection rates and school closures at a granular level (for example, municipality or school district) in real time (for example, daily) to inform their policy decisions.

## Results

### Data

In our study, the unit of observation is a municipality. In Japan, each municipality is responsible for the closures of its elementary (K1–6, students aged 6 to 12 years) and junior high (K7–9, students aged 12 to 15 years) schools (on average, 13.5 and 6.9 schools per municipality, respectively). In principle, children who attend public schools do so in the same municipality in which they reside. School closures began on 2 March 2020. The Ministry of Education, Culture, Sports, Science and Technology (MEXT) conducted municipal-level surveys on whether schools were open eight times in 2020: 4 and 16 March; 6, 10, 16 and 22 April; 11 May; and 1 June (Methods). For each survey date, the treatment variable is equal to one if all elementary and junior high schools in the municipality are closed as of the survey date and zero if they are open. When some schools are closed but others are open, the treatment variable has a missing value.

**Extended Data Fig. 1:**
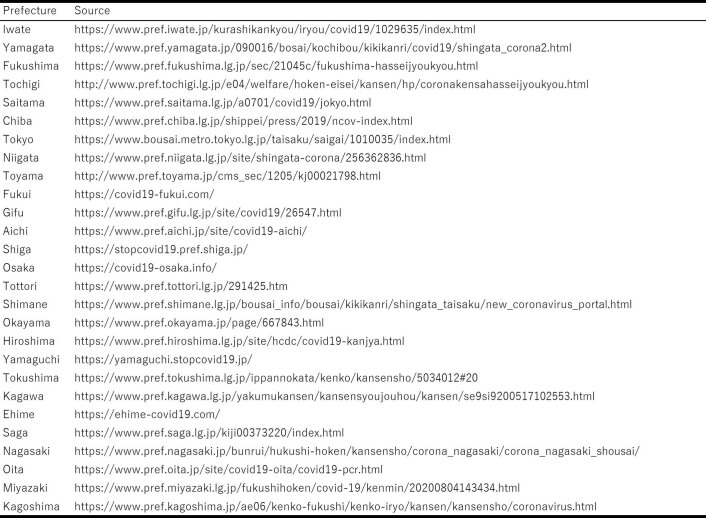
Sources of the daily number of newly confirmed cases: analyzed prefectures. The daily number of newly confirmed cases was collected from these prefectures’ websites on 12 August 2020. The URLs (but not the data) were updated on 22 March 2021. Source data

The outcome variables are the daily numbers of newly confirmed cases of COVID-19 per 100,000 residents of the municipality. Due to the availability of the outcome variables, we only use data from 26 (for 6 April and earlier treatments) or 27 (for 10 April and later treatments) of the 47 prefectures in Japan (Methods and Extended Data Fig. 1).

**Extended Data Fig. 2:**
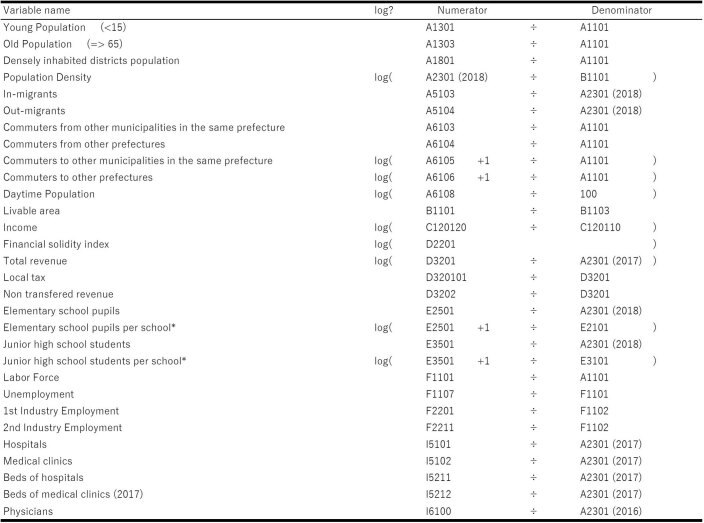
Definition of covariates. Numerators and denominators refer to the variable numbers in National Statistics Center, ‘Tokei de miru todofuken shichoson no sugata (shakai jinko tokei taikei) [System of social and demographic statistics (municipality data)]’ https://www.e-stat.go.jp/regional-statistics/ssdsview/municipality (accessed on October 5 and 15, 2020 and February 9, 2021). The year in parenthesis next to the variable A2301 refers to as of when the variable refers to the population. The value of the two variables with asterisk * is equal to zero when the denominator takes the value of zero. Source data

To address confounding, we controlled for dozens of covariates (Methods). First, we controlled for school closure status as of each past survey date, the total number of COVID-19 cases before the survey date and the number of COVID-19 cases in each of the past 7 d. We also included a set of 25 or 26 prefecture dummy variables, each of which indicates whether a given municipality belongs to the corresponding prefecture, to account for variation across prefectures. In Japan, prefectures have the primary responsibility for public health, including infectious disease. The governor of a prefecture can issue (de jure or de facto) NPI requests such as stay-at-home warnings, business closures and event suspensions. Because these NPIs are requests, not orders, citizens have no legal obligation to obey such NPIs. Thus, by including prefecture dummies, we could account for any characteristics of prefectures, including NPIs, which differed across prefectures during the time of our study. As a result, there was no remaining variation in NPIs among municipalities other than school closures. For this reason, we believe that our research design can identify the effect of school closures. We also controlled for latitude and longitude so that closer municipalities are more likely to be matched. Additionally, we controlled for 39 social, economic and political variables (Methods and Extended Data Fig. 2).

There are 1,741 municipalities in Japan. This number was reduced to 785 or 847 when we limited our analysis to the 26 or 27 prefectures mentioned above. Moreover, if any variable had a missing value in an analysis for a treatment variable, that municipality was not used in the analysis for the treatment variable. For details of our variables, see Methods. Table 2 presents the number of treated and control municipalities by survey date. Below, our analysis covers up to the 11 May survey date (Methods).

**Table 2:** Number of treated (closed schools) and control (open schools) municipalities by survey date

Survey date	Treated	Control	All	Treated (%)
4 March	761	10	771	98.7
16 March	718	29	747	96.1
6 April	256	483	739	34.6
10 April	491	307	798	61.5
16 April	523	267	790	66.2
22 April	710	80	790	89.9
11 May	641	145	786	81.6
1 June	2	783	785	0.3

### Matching

It is challenging to estimate causal effects from observational data rather than experimental data mainly because it is hard to control for confounders. Matching is one commonly used method to address these problems^27,50^. Here, for every control municipality, we matched a treated municipality that had similar values across our covariates to those of the control municipality (Methods). The average of the matched treated municipalities’ outcomes can identify the average of the counterfactual outcomes for the control municipalities if they were assigned treatment. Therefore, by subtracting the average of the control municipalities from that of the matched treated municipalities, we can estimate the average treatment effect on the control (ATC)^50^, namely, the degree to which school closures would change the number of COVID-19 cases in municipalities that actually did not close their schools (Methods). That is, if school closures have a causal effect to reduce the number of cases, the ATC should be negative. Note that most empirical studies (implicitly) assume some type of model (such as a linear model), while our identification strategy does not.

Recall that we do not know whether municipalities closed or opened their schools between the survey dates. In addition, most previous work focused on 7 to 14 d after implementation of an NPI, considering periods of incubation, test and report delay^13^. Accordingly, we cannot conduct panel data analysis from March to June 2020. Instead, for every set of survey date and outcome date, we implemented a cross-sectional analysis across municipalities where we performed matching and regressed the outcome variable on the treatment variable using only the matched municipalities.

### Main analysis

**Fig. 1:**
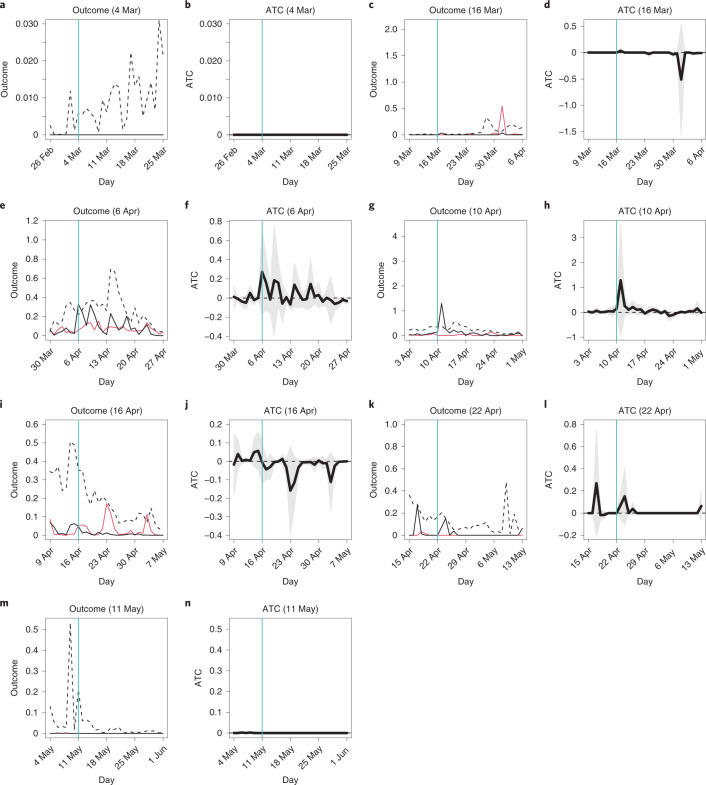
Average outcomes and ATC values: main analysis. **a**–**n**, The horizontal axis indicates dates in 2020, and the vertical turquoise line corresponds to the survey date. In **a**, **c**, **e**, **g**, **i**, **k** and **m**, the vertical axis represents the average number of confirmed cases per 100,000 residents, the black and red lines correspond to the average outcomes of the matched treated and control municipalities, respectively, and the dashed black line represents the average outcomes of all treated municipalities, both matched and unmatched. In **b**, **d**, **f**, **h**, **j**, **l** and **n**, the vertical axis represents ATC values, the thick black line indicates the point estimates of ATC values, and the shaded gray area presents the 95% confidence intervals. Cluster-robust standard errors were utilized where clusters were a pair of matched municipalities and a municipality. **a**, Outcome, 4 March. **b**, ATC, 4 March. **c**, Outcome, 16 March. **d**, ATC, 16 March. **e**, Outcome, 6 April. **f**, ATC, 6 April. **g**, Outcome, 10 April. **h**, ATC, 10 April. **i**, Outcome, 16 April. **j**, ATC, 16 April. **k**, Outcome, 22 April. **l**, ATC, 22 April. **m**, Outcome, 11 May. **n**, ATC, 11 May. Source data

Figure 1 presents the results. Each panel corresponds to either the average outcomes (number of COVID-19 cases per 100,000 municipal residents) or ATC values for the treatment variable (school closure status) as of a survey date. As an example, we will focus on the 6 April treatment variable (Supplementary Information; Main analysis, 6 April). In Fig. 1e, the horizontal axis indicates the dates of outcome variables, and the vertical axis represents the values of outcome variables. We examine 7 d before the survey date to 21 d after the survey date. The vertical turquoise line marks the survey date. The red line corresponds to the average outcome values of the control municipalities. The dashed black line shows the average outcome values of all treated municipalities, both matched and unmatched. If we compare the number of COVID-19 cases between the control (red line) and treated (dashed black line) municipalities without taking matching into account, the latter group has a higher number of cases than the former every day. But these two groups are not comparable. When we pay attention to the period before the survey date, the average number of cases in both groups follows distinct trajectories. This may suggest that control municipalities kept their schools open exactly because, in consideration of school closures, they had fewer cases, and thus they were more confident that they would be free from COVID-19 than treated municipalities.

To address concerns such as confounders and/or reverse causality, we shift to the solid black line, which indicates the average outcome values of the matched treated municipalities. Now, because both treated and control group averages almost overlap before the survey date, we can see that the matched groups share a similar infection history. This test is similar to the parallel trend check in the differences-in-differences technique^46^. Moreover, the differences in other covariates between the treated and control groups were also much smaller after matching than before (Supplementary Fig. 1 and Supplementary Table 3). Therefore, differences between the matched groups cannot be attributed to previous levels of infection or any other covariates.

Even after the survey date, the average outcomes of the matched treated municipalities are for the most part not smaller than those of the control municipalities, including 1–2 weeks after the survey date, the period that is the focus of most studies. But recall that the matched treated municipalities closed their schools even though they were in a similar situation to the control municipalities. If school closures have an effect to reduce the spread of COVID-19, the matched treated municipalities would have fewer cases than the control municipalities (or the control municipalities would fail to prevent an outbreak and have more cases). Instead, we find few significant differences in case numbers between the matched treated and control municipalities.

Correspondingly, the ATC values are almost null. In Fig. 1f, the vertical axis represents the estimates of the ATC values, where the thick black line indicates the point estimates, and the shaded gray area presents the 95% confidence intervals (Methods). Note that by subtracting the average outcome values of the control municipalities (red line in Fig. 1e) from those of the matched treated municipalities (black line in Fig. 1e), we obtained the ATC point estimates (thick black line in Fig. 1f). The ATC values were rarely significantly negative. Therefore, we cannot say that the school closures treatment as of 6 April had a causal effect on reducing the number of cases.

Other panels of Fig. 1 show the average outcomes and ATC values of other treatment variables in a similar fashion (Supplementary Information; main analysis). The implications of our findings for 6 April also hold for the remaining survey dates: school closures did not significantly reduce the spread of COVID-19 in Japan between 4 March and 1 June 2020. For some irregular spikes of ATC values, see the Supplementary Information (main analysis, 16 March)

### Robustness checks

To confirm that the above results are robust, we conducted a battery of robustness checks.

#### Controlling for covariates

**Fig. 2:**
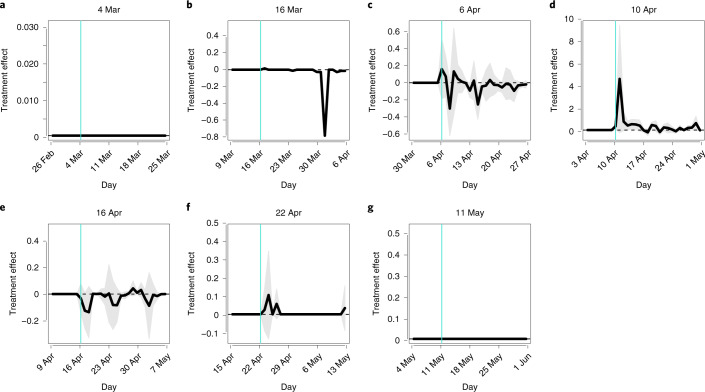
Treatment effects: controlling for covariates. **a**–**g**, The horizontal axis indicates dates in 2020, and the vertical axis represents the coefficient estimates of the treatment variable. The vertical turquoise line corresponds to the survey date. The thick black line indicates the point estimates of the treatment variable’s coefficients, and the shaded gray area presents the 95% confidence intervals. Cluster-robust standard errors are utilized where clusters were a pair of matched municipalities and a municipality. **a**, 4 March. We did not draw the shaded gray area because the estimation procedure did not return the standard errors. **b**, 16 March. **c**, 6 April. **d**, 10 April. **e**, 16 April. **f**, 22 April. **g**, 11 May. Source data

By matching, we reduced the differences in covariates between treated and control groups, although we failed to remove them altogether (Supplementary Fig. 1). Following recommendations in ref. ^51^, we regressed the outcomes not only on a treatment but also on the covariates that we matched on to further decrease the effects of confounders. Each panel of Fig. 2 shows the coefficient estimates of the corresponding treatment variable. The ATC values again suggest that school closures do not lower the infection of SARS-CoV-2 significantly.

#### Negative binomial regression

**Extended Data Fig. 3:**
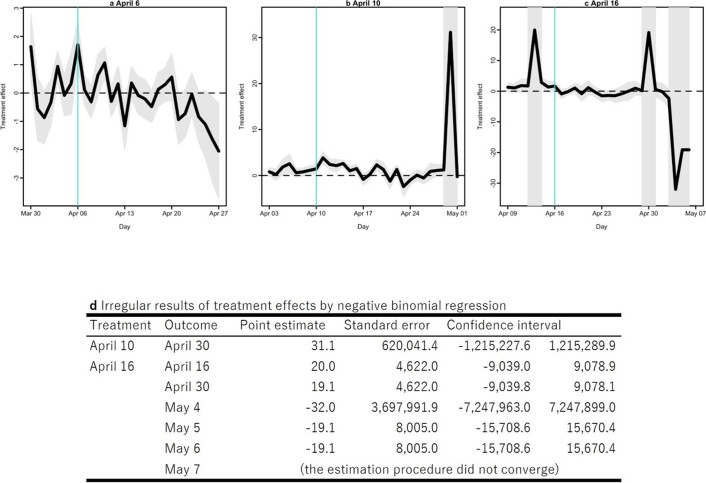
Treatment effects: negative binomial regression. In each panel, the horizontal axis indicates dates in 2020, and the vertical axis represents the coefficient estimates of the treatment variable. The vertical turquoise line corresponds to the survey date. The thick black line indicates the point estimates of the treatment variable’s coefficients, and the shaded grey area presents the 95% confidence intervals. Cluster-robust standard errors are utilized where clusters are a pair of matched municipalities and a municipality. **a**, April 6. **b**, April 10. **c**, April 16. **d**, Irregular results of treatment effects by negative binomial regression. For other survey dates, we cannot estimate negative binomial regression parameters. Source data

Because the numbers of cases are nonnegative integers (Supplementary Fig. 2), some studies rely on negative binomial regression^21,22,41^. Therefore, we also applied negative binomial regression to our matched data^52^, using the log of the population size as an offset variable so that various sized municipalities are comparable. Unfortunately, the estimation procedures only converged for the treatment variables as of 6, 10 and 16 April, and only if we did not include any covariates. The coefficient estimates of the treatment variables sometimes implied that school closures even significantly increased the number of cases, while they rarely (significantly) decreased the number of cases (Extended Data Fig. 3). The findings remain the same: there is no evidence that school closures reduce the number of COVID-19 cases.

#### Public health center fixed effects

**Fig. 3:**
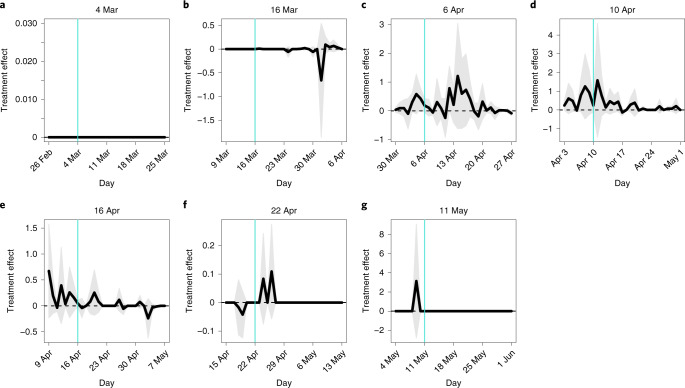
Treatment effects: public health center fixed effects. **a**–**g**, The horizontal axis indicates dates in 2020, and the vertical axis represents the coefficient estimates of the treatment variable. The vertical turquoise line corresponds to the survey date. The thick black line indicates the point estimates of the treatment variable’s coefficients, and the shaded gray area presents the 95% confidence intervals. Cluster-robust standard errors are utilized where clusters were a public health center. **a**, 4 March. **b**, 16 March. **c**, 6 April. **d**, 10 April. **e**, 16 April. **f**, 22 April. **g**, 11 May. Source data

Public health centers in Japan are in charge of implementing public health policy, including counting COVID-19 cases. There are a total of 263 public health centers in the 27 prefectures in this study, each covering 1 to 13 municipalities (Supplementary Fig. 3). To address heterogeneity in the centers’ efforts at confirming and/or tracking cases, we regressed each of our outcome variables on one of our treatment variables and public health center dummy variables, using both matched and unmatched municipalities. We identified the effect of the treatment variable by (a weighted average of) the difference in means of the outcome variable between treated and control municipalities within a public health center (Supplementary Table 5). Because a public health center covers adjacent municipalities, its fixed effect controls for any idiosyncratic factors not only held by the public health center itself but also shared by the municipalities it covers (for example, urbanicity), effectively substituting for many covariates we did not technically control for. Figure 3 illustrates the coefficient estimates of the treatment variables. Again, we did not find in this analysis that school closures have significant effects on the number of COVID-19 cases.

#### Inverse probability weighting

**Extended Data Fig. 4:**
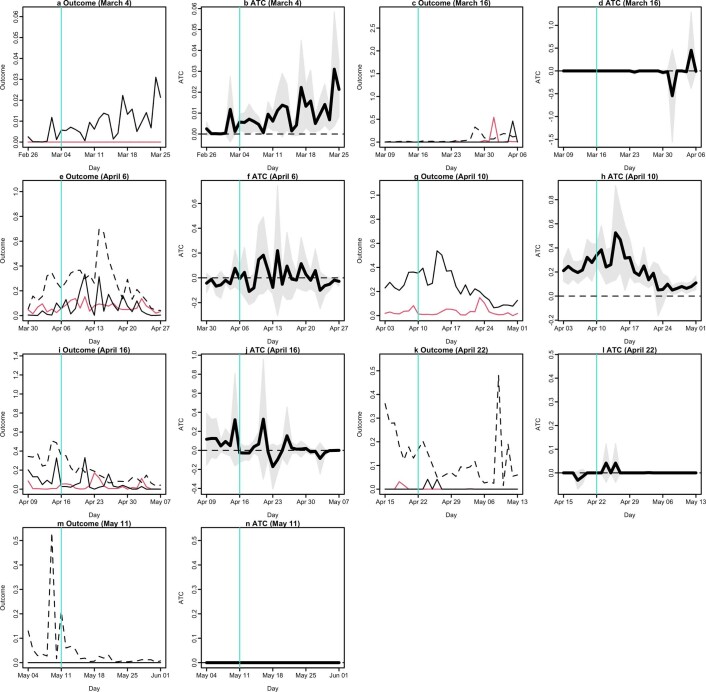
Average outcomes and ATCs: inverse probability weighting. In each panel, the horizontal axis indicates dates in 2020, and the vertical turquoise line corresponds to the survey date. In the outcome panels (**a**, **c**, **e**, **g**, **i**, **k**, **m**), the vertical axis represents the average number of confirmed cases per 100,000 residents, the black and red lines correspond to the weighted average outcomes of the treated and control municipalities, respectively, and the dotted black line represents the un-weighted average outcomes of treated municipalities. In the ATC panels (**b**, **d**, **f**, **h**, **j**, **l**, **n**), the vertical axis represents ATCs, the thick black line indicates the point estimates of ATCs, and the shaded grey area presents the 95% confidence intervals. We calculate standard errors, taking into consideration weight. **a**, outcome, March 4. The dotted black line overlaps the solid black line and is thus invisible. **b**, ATC, March 4. **c**, outcome, March 16. **d**, ATC, March 16. **e**, outcome, April 6. **f**, ATC, April 6. **g**, outcome, April 10. The dotted black line overlaps the solid black line and is thus invisible. **h**, ATC, April 10. **i**, outcome, April 16. **j**, ATC, April 16. **k**, outcome, April 22. **l**, ATC, April 22. **m**, outcome, May 11. **n**, ATC, May 11. Source data

An alternative to matching is inverse probability weighting^20^ (Methods). When we estimated the ‘probability’ (that is, propensity score and thus weight), we used the same covariates as in matching except latitude and longitude. By subtracting the average outcome of the control municipalities from the weighted average of the treated municipalities (Extended Data Fig. 4a,c,e,g,i,k,m), we could estimate the ATC (Extended Data Fig. 4b,d,f,h,j,l,n). The ATC values suggest that municipalities that closed their schools mostly increased the number of cases (Supplementary Information; robustness checks, inverse probability weighting) and did not significantly decrease the spread of COVID-19 for the vast majority of dates. The results further support our main findings.

#### Conditioning on neighbors

Currently, most causal inference studies assume that the outcome of a municipality is independent of other municipalities’ treatments, which is implied by the stable unit treatment value assumption^50^. But in our context, the treatment and outcome in one municipality will affect those in another^46^. It is challenging to take into consideration such spatial spillover.

One state-of-the-art study proposes the stable unit treatment on neighborhood value assumption: the outcome of a municipality is independent of other municipalities’ treatments conditioned on (the average of) the treatments of neighboring municipalities^53^. For all of the survey dates except 6 April, the most frequent pattern was the situation where a municipality was surrounded by only treated municipalities (on 6 April, this situation was the second most frequent; Supplementary Table 6 and Supplementary Fig. 4); therefore, we estimated the ATC value given all neighbor municipalities are treated by limiting our analyses to only those municipalities whose neighboring municipalities were all treated. We applied matching to those municipalities. Because this condition is restrictive, we only ended up with two to six pairs of matched municipalities for each survey date (Supplementary Table 7). Thus, we warn that the results may not be reliable. For the treatment on 10 April, there remained only one control municipality, and the matching algorithm failed to match any treated municipality to it; therefore, we did not report the corresponding results.

**Fig. 4:**
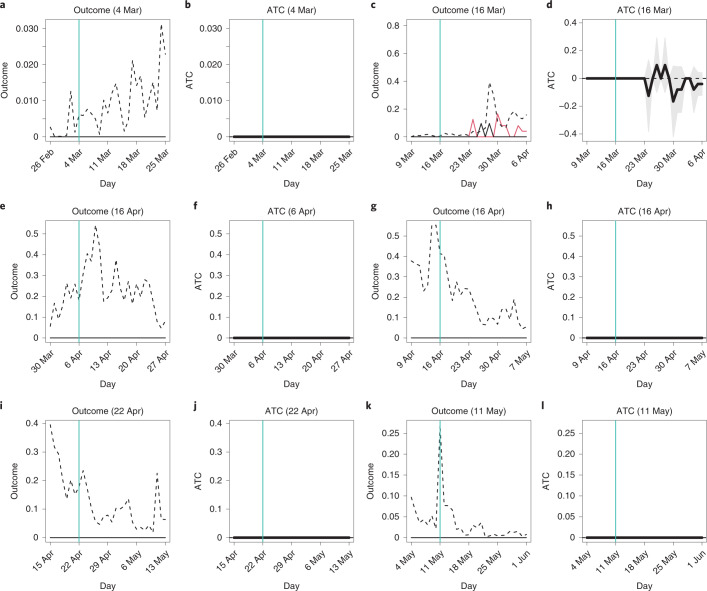
Average outcomes and ATC values: conditioning on neighbors. **a**–**k**, the horizontal axis indicates dates in 2020, and the vertical turquoise line corresponds to the survey date. In **a**, **c**, **e**, **g**, **i** and **k**, the vertical axis represents the average number of confirmed cases per 100,000 residents, the black and red lines correspond to the average outcomes of the matched treated and control municipalities, respectively, and the dashed black line represents the average outcomes of all treated municipalities, both matched and unmatched. In **b**, **d**, **f**, **h**, **j** and **l**, the vertical axis represents ATC values, the thick black line indicates the point estimates of ATC values, and the shaded gray area presents the 95% confidence intervals. Cluster-robust standard errors are utilized where clusters were a pair of matched municipalities and a municipality. **a**, outcome, 4 March. **b**, ATC, 4 March. **c**, Outcome, 16 March. **d**, ATC, 16 March. **e**, Outcome, 6 April. **f**, ATC, 6 April. **g**, Outcome, 16 April. **h**, ATC, 16 April. **i**, Outcome, 22 April. **j**, ATC, 22 April. **k**, Outcome, 11 May. **l**, ATC, 11 May. Source data

As shown in Fig. 4, for most days, treated and control municipalities had no cases. Therefore, the ATC values imply that school closures do not change the number of cases, although they either increase or decrease the number of cases, albeit insignificantly, on several days. If opening schools leads to the spread of COVID-19, spikes of cases would occur in the control group; however, these were not observed. The implication is the same: school closures do not help reduce the spread of COVID-19 significantly.

#### Average treatment effect on the treated

**Fig. 5:**
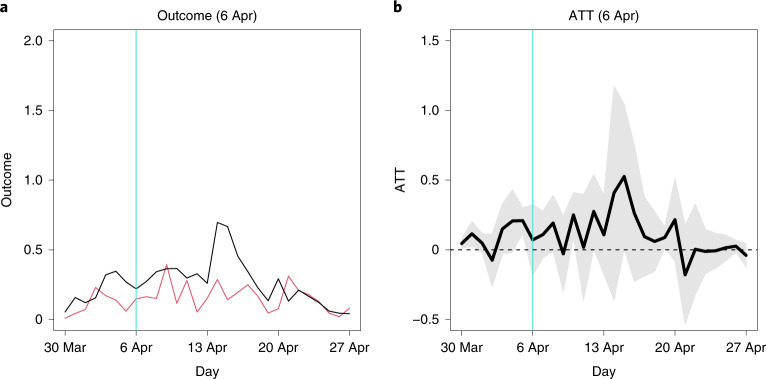
Average outcomes and ATT values. **a**,**b**, The horizontal axis indicates dates in 2020. The vertical turquoise line corresponds to the survey date, 6 April. **a**, The vertical axis represents the average number of confirmed cases per 100,000 residents. The black and red lines correspond to the average outcomes of the matched treated and control municipalities, respectively. **b**, The vertical axis represents ATT values. The thick black line indicates the point estimates of ATTs, and the shaded gray area presents the 95% confidence intervals. Cluster-robust standard errors are utilized where clusters were a pair of matched municipalities and a municipality. Source data

We next estimated the average treatment effect on the treated (ATT), namely, the degree to which school closures changed the number of COVID-19 cases in municipalities that actually did close their schools compared to how many cases those same municipalities would have if their schools were instead open (Methods). To estimate an ATT, we compare a sufficiently similar control municipality to every treated municipality. Because only the 6 April survey date had more control municipalities than treated ones (Table 2), we only estimated the ATT values for the 6 April treatment variable. Compared with the matched control municipalities (red line in Fig. 5a), the (matched) treated municipalities (black line in Fig. 5a) had similar average outcomes before the survey date and mostly larger average outcomes after the survey date. (In Fig. 5a, the dashed black line (all of the treated municipalities) overlaps the solid black line and is thus invisible because all of the treated municipalities are matched to control municipalities.) Accordingly, the ATT values never indicate that school closures significantly reduced local COVID-19 cases (Fig. 5b). Therefore, our null findings apply not only for ATC values but also ATT values, at least for the 6 April treatment.

#### A smaller set of covariates

**Extended Data Fig. 5:**
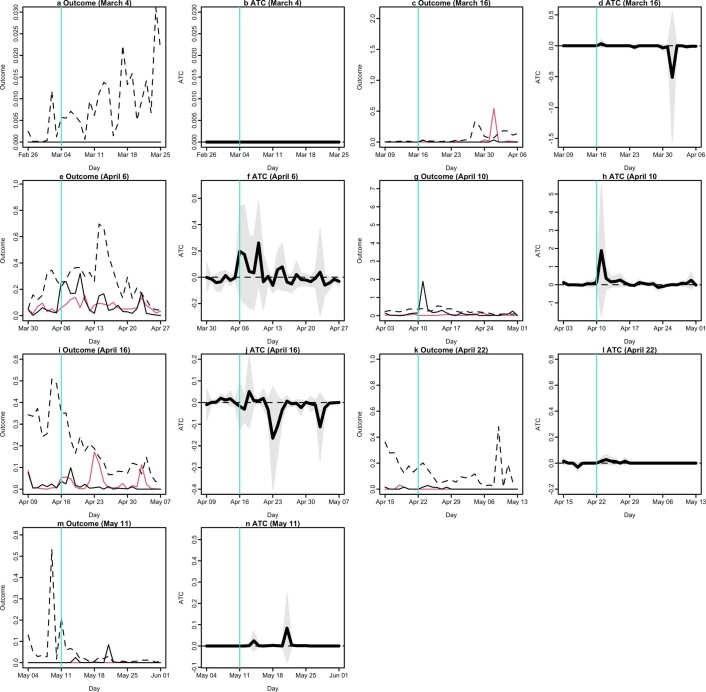
Average outcomes and ATCs: a smaller set of covariates. In each panel, the horizontal axis indicates dates in 2020, and the vertical turquoise line corresponds to the survey date. In the outcome panels (**a**, **c**, **e**, **g**, **i**, **k**, **m**), the vertical axis represents the average number of confirmed cases per 100,000 residents, the black and red lines correspond to the average outcomes of the matched treated and control municipalities, respectively, and the dotted black line represents the average outcomes of all treated municipalities, both matched and unmatched. In the ATC panels (**b**, **d**, **f**, **h**, **j**, **l**, **n**), the vertical axis represents ATCs, the thick black line indicates the point estimates of ATCs, and the shaded grey area presents the 95% confidence intervals. Cluster-robust standard errors are utilized where clusters are a pair of matched municipalities and a municipality. **a**, outcome, March 4. **b**, ATC, March 4. **c**, outcome, March 16. **d**, ATC, March 16. **e**, outcome, April 6. **f**, ATC, April 6. **g**, outcome, April 10. **h**, ATC, April 10. **i**, outcome, April 16. **j**, ATC, April 16. **k**, outcome, April 22. **l**, ATC, April 22. **m**, outcome, May 11. **n**, ATC, May 11. Source data

Because we matched on dozens of covariates, readers may be concerned about potential over-adjusting for confounding and collider bias. To address this concern, in the setup of the main analysis, we matched on only 16 of the 39 social, economic and political variables in addition to the past treatment variables, the set of prefecture dummies and eight outcomes (the total number of COVID-19 cases before the survey date and the number of COVID-19 cases in each of the past 7 d; Methods). Again, our results fail to confirm that school closures decrease the number of COVID-19 cases (Extended Data Fig. 5).

## Discussion

We identify the effect of school closures on the spread of COVID-19 using matching methods. Our results suggest that the effect of school closures on COVID-19 cases in Japan in early 2020 is not significantly different from zero. Ref. ^45^ comes to the same conclusion based on different data from Japan and a different method.

Our research design does not enable us to elucidate the mechanisms underlying the empirical findings. That said, we provide a few conjectures. First, many treated municipalities did not comply with the treatment in the sense that many schools that were closed nevertheless provided spaces for children whose parents worked such as playing fields, gyms, classrooms and libraries. For instance, schools made these facilities available to families in 63.6% of the treated municipalities as of 17 March and 59% as of 16 April (Supplementary Information; main analysis, 16 March and 16 April). Furthermore, children may interact with each other outside of school^19,26,36,41^. Second, as past studies have demonstrated^19,36,40,54^, even in control municipalities, children may be less susceptible to SARS-CoV-2 and less likely to transmit the virus to others, including teachers, parents and neighbors. Moreover, students attending in-person schooling were engaged in stringent mitigation strategies such as physical distancing and enhanced hygiene. Limiting the amount of contact between children by shutting down schools may thus have not had a large impact on reducing the spread of COVID-19.

We admit some limitations of our study. Although we are confident in the internal validity of our findings, we are less sure about their external validity (Supplementary Information). Japan, like New Zealand and South Korea, fared relatively well with regard to COVID-19 cases compared to some countries such as Italy, Spain and the United States. It is possible that school closures only have a discernible effect on COVID-19 cases once the reproduction number (within a school environment) passes a certain threshold. Likewise, we analyze the effectiveness of school closures in the initial months of the coronavirus pandemic, but community transmission has become notably worse in many countries (including Japan) since spring 2020. New variants of SARS-CoV-2 (for example, the alpha and delta variants), which have each increased transmissibility, may also be more or less affected by school closures. Another possibility is that citizens’ behavior (for example, social distancing, wearing masks and washing hands) and school measures (for example, disinfecting classrooms, placing transparent dividers between students, splitting classes into smaller groups that meet separately and ventilation) intended to limit the spread of COVID-19 may be less strict in some countries compared to Japan, which could increase the risk of opening schools.

With these caveats in mind, we offer both empirical and methodological contributions to the existing literature. Empirically, we find no evidence that school closures in Japan caused a significant reduction in the number of coronavirus cases. These null results concerning the supposed benefits of closing schools suggest that policymakers should be cautious when considering similar policies in the future, especially given the substantial costs such policies can have for the well-being of both children and parents. Our recommendation is that governments should monitor SARS-CoV-2 infection rates and school closures at a granular level (for example, municipality or school district) in real time (for example, daily). Methodologically, we pay attention to causal inference by using matching techniques and exploiting features of Japanese municipalities that enable us to identify the effect of school closures independent of other NPIs. Our hope is that our study can inspire other researchers to use causal inference methods to rigorously test the generalizability of our findings to other settings.

## Methods

### Data

#### Treatment variables

We focused on schools established by municipalities. Usually, each school is established by one municipality. However, sometimes a few municipalities jointly establish a ‘school authority’ and operate the school together. In such a case, we regarded the school as belonging to each of the municipalities. The sources that we used from the MEXT treat the ‘founder’ of schools as the unit of observation, that is, a municipality and a school authority.

Most of the schools we studied are elementary schools (K1–6) and junior high schools (K7–9). We excluded high schools (K10–12, most of which are established by prefectures), universities (most of which are established by the national government and private entities) and nurseries.

The MEXT conducted surveys on school closures across municipalities nine times in 2020. (Besides the websites we refer to below, see the MEXT, ‘shingata korona uirusu ni kanrenshita kansensho taisaku ni kansuru taio ni tsuite: shinchaku joho (on the measures to address COVID-19: news),’ https://www.mext.go.jp/a_menu/coronavirus/index_00012.html; accessed on 17 June 2021). We collapsed two of them into the 16 March survey as detailed below. If a survey does not report whether all or some of schools are closed in a municipality (for example, the school authority that the municipality is part of did not respond to the survey), we coded the treatment variable as having a missing value in the municipality.

##### 4 March

The source is the MEXT, ‘shingata korona uirusu kansensho taisaku no tame no sho-chu-koto gakkoto ni okeru rinji kyugyo no jokyo ni tsuite (on the situation of temporary closures of elementary, junior high and high schools to address COVID-19),’ 4 March 2020 (https://www.mext.go.jp/content/20200304-mxt_kouhou02-000004520_1.pdf; accessed on 7 August 2020). The survey lists municipalities that opened all of their elementary and/or junior high schools. We supposed that other municipalities and school authorities closed all of their elementary and junior high schools.

##### 16 March

The sources are the MEXT, ‘shingata korona uirusu kansensho taisaku no tame no shogakko chugakko kotogakko oyobi tokubetsushien gakkoto ni okeru rinji kyugyo no jokyo ni tsuite (on the situation of temporary closures of elementary, junior high, high and special education schools to address COVID-19),’ 16 March 2020 (https://www.mext.go.jp/content/20200316_mxt_kouhou02_000004520-1.pdf; survey 1, accessed on 7 August 2020) and the MEXT, ‘shingata korona uirusu kansensho taisaku no tame no shogakkoto no rinji kyugyo ni kanrenshita kodomo no ibasho no kakuhoto ni kansuru kaku jichitai no torikumi jokyo ni tsuite (on the situation of each municipality’s measure to prepare for the children’s place concerned with temporary closures of elementary and other schools to address COVID-19),’ 17 March 2020 (https://www.mext.go.jp/content/20200317-mxt_kouhou02-000004520_1.pdf; survey 2, accessed on 7 August 2020). Survey 1 lists the founders that planned to open all of their elementary and/or junior high schools as of 16 March. Survey 2 lists the founders that were not listed in survey 1 and ended closures of their elementary and/or junior high schools by 15 March. In sum, we can say that both groups of founders opened all of their schools as of 16 March. We supposed that other founders closed all of their schools as of 16 March. Hamamatsu City has a missing value because surveys 1 and 2 contradict each other.

##### 6 April

The source is the MEXT, ‘shingata korona uirusu kansensho taisaku ni kansuru gakko no shingakki kaishi jokyoto ni tsuite (on the situation of the beginning of schools’ new term concerned with measures to address COVID-19),’ 6 April 2020 (https://www.mext.go.jp/content/20200407-mxt_kouhou01-000006421_1.pdf; accessed on 29 June 2020). The survey lists the founders that closed their schools. We supposed that other founders opened all of their schools.

##### 10 April

The source is the MEXT, ‘shingata korona uirusu kansensho taisaku ni kansuru gakko no shingakki kaishi jokyoto ni tsuite (on the situation of the beginning of schools’ new term concerned with measures to address COVID-19),’ 10 April 2020 (https://www.mext.go.jp/content/20200413-mxt_kouhou01-000006421_1.pdf; accessed on 29 June 2020). The survey lists founders that closed their schools. We supposed that other founders opened all of their schools.

##### 16 April

The source is the MEXT, ‘gakushu shidoto torikumi jokyo chosa (survey on the status of learning guidance),’ 16 April 2020. We obtained the source data files from the MEXT on 24 December 2020 with their permission. The aggregated results are reported at the MEXT, ‘shingata korona uirusu kansensho taisaku no tame no gakko no rinji kyugyo ni kanrenshita koritsu gakko niokeru gakushu shidoto no torikumi jokyo ni tsuite (on the status of learning guidance in public schools concerned with temporary closures of schools to address COVID-19),’ 16 April 2020 (https://www.mext.go.jp/content/20200421-mxt_kouhou01-000004520_4.pdf; accessed on 29 October 2020). The MEXT asked founders to respond if they closed their schools. Thus, we supposed that founders who did not respond opened all of their schools.

##### 22 April

The source is the MEXT, ‘rinji kyugyo jisshi jokyo chosa (survey on the status of temporary school closures),’ 22 April 2020. We obtained the source data files from the MEXT on 24 December 2020 with their permission. The aggregated results are reported at the MEXT, ‘shingata korona uirusu kansensho taisaku no tame no gakko niokeru rinji kyugyo no jisshi jokyo ni tsuite (on the status of temporary closures of schools to address COVID-19),’ April 22, 2020 (https://www.mext.go.jp/content/20200424-mxt_kouhou01-000006590_1.pdf; accessed on 14 October 2020). As for surveys after 22 April, the source data files that the MEXT provides clarify the information not only about elementary and junior high schools but also about ‘obligatory education schools’ (K1– 9), ‘secondary education schools’ (K7–12) and ‘special education schools’ (K1–12 and preschool for those who have disabilities and need special help), all of which we take into account in making the treatment variables. In principle, all founders are supposed to respond, although in practice, some failed to do so and their treatment variable (or the treatment variable of municipalities whose school authorities failed to respond) has a missing value due to data unavailability.

##### 11 May

The source is the MEXT, ‘rinji kyugyo jisshi jokyo chosa (survey on the status of temporary school closures),’ 11 May 2020. We obtained the source data file from the MEXT on 24 December 2020 with their permission. The aggregated results are reported at the MEXT, ‘shingata korona uirusu kansensho taisaku no tame no gakko ni okeru rinji kyugyo no jisshi jokyo ni tsuite (on the status of temporary closures of schools to address COVID-19),’ 11 May 2020 (https://www.mext.go.jp/content/20200513-mxt_kouhou02-000006590_2.pdf; accessed on 14 October 2020). One municipality has a missing value because its answer is inconsistent.

##### 1 June

The source is the MEXT, ‘gakko saikai jokyo chosa (survey on the status of school reopenings),’ 4 June 2020. We obtained the source data file from the MEXT on 24 December 2020 with their permission. The aggregated results are reported at the MEXT, ‘shingata korona uirusu kansensho ni kansuru gakko no saikai jokyo ni tsuite (on the status of reopening of schools concerned with COVID-19),’ 1 June 2020 (https://www.mext.go.jp/content/20200603-mxt_kouhou01-000004520_4.pdf; accessed on 14 October 2020).

#### Outcome variables

Our outcome variables were the daily number of newly confirmed cases per 100,000 residents. We collected the daily number of newly confirmed cases from prefectures’ websites, whose URLs are displayed in Extended Data Fig. 1 and Supplementary Table 1 (accessed on 12 August 2020, although we updated the URLs (but not the data) on 22 March 2021; an exception is Tokyo Prefecture, whose URL was accessed on 15 January 2021). The values of the outcome variables were only available for all municipalities in 26 of 47 prefectures. In addition, we coincidentally obtained municipal-level data from Tokushima Prefecture through a Freedom of Information Act (FOIA) request. Therefore, we only analyzed 27 ‘target’ prefectures.

We were careful when analyzing data from Tokyo Prefecture. The prefecture only started reporting the cumulative number of confirmed cases in each of its 62 municipalities after 31 March. By taking the difference between two consecutive days, we were able to obtain the daily number of newly confirmed cases, but only after 1 April. It is worth noting that in 17 observations, these differences are equal to −1, perhaps because Tokyo corrected the data from the day before; we changed these values to zero. Accordingly, we were able to calculate these values for the past 7 d only after 8 April. Therefore, Tokyo prefecture was included only for the treatments as of 10 April and after. This is why the number of target prefectures is 26 for 6 April and earlier treatments and 27 for 10 April and later treatments.

Data on population (including foreigners) is as of 1 January 2020 (latest available data). The source is ‘jumin kihon daicho ni motoduku jinko, jinko dotai oyobi setaisu (population, its movement and the number of households based on residential basic book),’ 1 January 2020 (https://www.soumu.go.jp/main_sosiki/jichi_gyousei/daityo/jinkou_jinkoudoutai-setaisuu.html; accessed on 27 January 2021).

Because the daily number of newly confirmed cases in a municipality was small and our study period (26 February to 1 June) was at an early stage of the pandemic, the number of deaths were much smaller and thus not analyzed. For the same reason, we cannot calculate the reproduction number.

#### Covariates

Besides the past treatment variables and the set of prefecture dummies, we used 49 covariates. They included: the sum of all the outcome variables before the survey date; the outcome variables from the past 7 d; the number of municipalities covered by the public health center that is in charge of a municipality; five demographic variables (population, population density, the young, the old and densely inhabited districts); seven commuting variables (in-migrants, out-migrants, commuters from other municipalities in the same prefecture, commuters from other prefectures, commuters to other municipalities in the same prefecture, commuters to other prefectures and daytime population); four geographic variables (inhabitable area size, the number of bordering municipalities, latitude and longitude); income; four variables on the municipal government’s fiscal situation (financial solidity index, total revenue, local tax and non-transferred revenue); four education variables (elementary school pupils, elementary school pupils per school, junior high school students and junior high school students per school); four labor variables (labor force, unemployment, primary industry employment and secondary industry employment); five medical variables (hospitals, medical clinics, beds of hospitals, beds of medical clinics and physicians); three climatic variables (precipitation, daylight hours and average temperature); and three mayoral variables (age, number of terms and days since last election). In the Supplementary Information (main analysis), we elaborate on why we chose these covariates. We divided the description of the covariates by their sources.

##### Government statistics

Extended Data Fig. 2 summarizes how to calculate most of the covariates. The numerators and denominators are original variables retrieved from the source, National Statistics Center, ‘tokei de miru todofuken shichoson no sugata (shakai jinko tokei taikei) (system of social and demographic statistics (municipality data))’ https://www.e-stat.go.jp/regional-statistics/ssdsview/municipality; accessed on 5 and 15 October 2020 and 9 February 2021). For the financial solidity index (D2201) of the 23 special wards in Tokyo Prefecture, we referred to Tokyo Metropolitan Government, Bureau of General Affairs, Local Administration Division, ‘tokubetsuku futsu kaikei kessan no jyokyo (the state of the settled general accounts of special wards), 2017’ (https://www.soumu.metro.tokyo.lg.jp/05gyousei/gyouzaisei/new/30tuika/29soukatsu-ku-shi/29gaiyo.xls; accessed on 8 February 2021). Below, the variables are explained and referred to by their official codes in the dataset (Supplementary Information; data, covariates, government statistics). The year for each variable is in parentheses. We refer to the latest available year. If the reference year is 2015, the original variable is in the census. If the unit is not the number of people or items, we mention it in the square brackets at the end of the line.

**A1101** Population (2015)

**A1301** Young population (14 and younger) (2015)

**A1303** Old population (65 and older) (2015)

**A1801** Densely inhabited districts population (2015)

**A2301** Population (2016–2018)

**A5103** In-migrants (2018)

**A5104** Out-migrants (2018)

**A6103** Workers and students to other municipalities in the same prefecture (2015)

**A6104** Workers and students to other prefectures (2015)

**A6105** Workers and students from other municipalities in the same prefecture (2015)

**A6106** Workers and students from other prefectures (2015)

**A6108** Daytime population relative to the population (percentage) (2015)

**B1101** Total land area (excluding the northern territories and Takeshima Island) (2018) [ha]

**B1103** Inhabitable area (2018) [ha]

**C120110** Taxable income (2018) [1,000 yen]

**C120120** Number of tax debtors (per income) (2018)

**D2201** Financial solidity index (2017)

**D3201** Total revenue (2017) [1,000 yen]

**D320101** Local tax revenue (2017) [1,000 yen]

**D3202** Non-transferred revenue (2017) [1,000 yen]

**E2101** Number of elementary schools (2018)

**E2501** Number of elementary school students (2018)

**E3101** Number of junior high schools (2018)

**E3501** Number of junior high school students (2018)

**F1101** Population in labor force (2015)

**F1102** Number of employed persons (2015)

**F1107** Number of unemployed persons (2015)

**F2201** Number of persons employed in primary industry (2015)

**F2211** Number of persons employed in secondary industry (2015)

**I5101** Number of hospitals (2017)

**I5102** Number of medical clinics (2017)

**I5211** Beds of hospitals (2017)

**I5212** Beds of medical clinics (2017)

**I6100** Number of physicians (2016)

We matched on neither census population in 2015 (the variable A1101) nor population in 2016–2018 (the variable A2301) but the latest population (including foreigners) as of 1 January 2020, which we introduced in the previous subsubsection. As for the variable A1801, we set missing values of this variable to zero, supposing that such municipalities have no densely inhabited districts. Other variables have no missing values.

In 13 municipalities in Fukushima Prefecture (Futaba, Hirono, Iitate, Iwaki, Katsurao, Kawamata, Kawauchi, Minami-soma, Namie, Naraha, Okuma, Tamura and Tomioka), which are eligible for the Nuclear Power Accident Evacuation Special Act, residents and students do not necessarily live in the municipalities. Therefore, we excluded these municipalities from our analysis.

##### Public health centers

Another covariate is the log of the number of municipalities that the public health center in charge of a given municipality is in charge of. The source is the Ministry of Health, Labor and Welfare, ‘todofuken betsu shikuchoson fugo oyobi hokenjo fugo (municipality codes and public health center codes by prefecture)’ (https://www.data.go.jp/data/dataset/mhlw_20170316_0002; accessed on 29 January 2021). We refer to this source when we use health center fixed effects as well. Although the ‘Matsue Public Health Center Established by Matsue City and Shimane Prefecture’ is assigned different public health center codes in Matsue and Yasugi Cities, which the center is in charge of, we supposed both cities had the same public health center. There are no missing values for this variable.

##### Meteorological data

We also matched on the annual normal values (average between 1981–2010) of precipitation (mm), daylight hours and average temperature (degrees Celsius). The source is ref. ^55^, which contains values of the three variables by 1-km-square mesh. We take their averages across 1-km-square grid cells in each municipality. A grid cell at the border of municipalities was assigned to the municipality that included the center of the grid cell. Ogasawara Village in Tokyo Prefecture, a far remote island, had missing values for these three meteorological variables and thus was excluded from our analysis.

##### Geographical data

We downloaded the shape files of all municipalities from the Ministry of Land, Infrastructure, Transport and Tourism, National Spatial Planning and Regional Policy Bureau, National Land Information Division, ‘kokudo suchi joho daunrodo (downloadable files of land numerical information)’ as of 1 January 2020 (https://nlftp.mlit.go.jp/ksj/index.html; accessed on 27 January 2021). Based on the files, we obtained latitude, longitude and the number of neighbor municipalities. We took the logarithm of the number of neighbor municipalities after adding one to it. Some municipalities (usually, islands) had no neighbor municipality. There were no missing values of these variables.

##### Mayoral data

We used data from Ichini, ‘senkyo dotto komu (election dot-com)’ (https://go2senkyo.com/; accessed on 28 April 2021) for information on mayors including their ages, number of terms and days since their last election. We measured them as of the survey date for each treatment variable. We filled in the few missing values of variables using newspaper coverage of elections.

### Matching

We implement genetic matching^56^ with replacement by using the MatchIt package^57^ in the statistical computing environment R^58^, which calls functions from the Matching package^59^. We matched on the covariates discussed in the previous section. Upon advice by ref. ^60^, as a metric of the distance between municipalities, we used the Mahalanobis distance. Following ref. ^59^, we set the arguments pop.size and nboots at 1,000. We kept other options at their default values.

### Statistical analyses: main analysis

To derive the confidence intervals of the ATC values, we utilized cluster-robust standard errors by using the lmtest and sandwich packages where clusters were a pair of matched municipalities and a municipality^61–63^.

### Statistical analyses: robustness checks

#### Negative binomial regression

We implemented negative binomial regression using the MASS package^64^.

#### Public health center fixed effects

Depending on the survey date, there were only 25 to 139 municipalities covered by public health centers that had responsibility for both treated and control municipalities, which contributed to the identification of the treatment effects (Supplementary Table 5). Therefore, we did not regress on any covariate. We implemented fixed-effects models using the lfe package^65^. Standard errors were clustered by public health center.

#### Inverse probability weighting

We estimated the propensity score using the covariate balancing propensity score algorithm^66^, which is implemented by the CBPS package. We estimated the ATC values by way of the WeightIt package^51,67^. When calculating standard errors, we considered weight by way of the survey package^68^.

#### Conditioning on neighbors

If any of the neighbor municipalities had a missing value of the treatment variable or if a municipality such as an island had no neighbor municipalities, such municipalities were excluded from the analysis. We considered neighbors outside the 27 target prefectures as well.

#### A smaller set of covariates

In addition to the past treatment variables and the set of prefecture dummies, we still matched on the following covariates: the total number of COVID-19 cases before the survey date; the number of COVID-19 cases in each of the past 7 d; the number of municipalities covered by the public health center that is in charge of a municipality; population; population density; the old; densely inhabited population districts; daytime population; income; financial solidity index; local tax; elementary school pupils; elementary school pupils per school; junior high school students; junior high school students per school; hospitals; medical clinics; and physicians.

### Reporting Summary

Further information on research design is available in the Nature Research Reporting Summary linked to this article.

## Online content

Any methods, additional references, Nature Research reporting summaries, source data, extended data, supplementary information, acknowledgements, peer review information; details of author contributions and competing interests; and statements of data and code availability are available at 10.1038/s41591-021-01571-8.

## Supplementary information

Supplementary Information Supplementary Text, Figs. 1–5 and Tables 1–10

Reporting Summary

Supplementary Data 1 Statistical source data for Supplementary Fig. 1

Supplementary Data 2 Statistical source data for Supplementary Fig. 2

Supplementary Data 3 Statistical source datafor Supplementary Fig. 3

Supplementary Data 4 Statistical source data for Supplementary Fig. 4

Supplementary Data 5 Statistical source data for Supplementary Fig. 5

## Data Availability

All data except the four surveys (as of 16 and 22 April, 11 May and 1 June 2020) from the MEXT are deposited to Harvard Dataverse as K.F., C.T.M. and K.N., 2021, ‘replication data for: no causal effect of school closures in Japan on the spread of COVID-19 in spring 2020,’ at 10.7910/DVN/N803UQ. All the data sources are detailed in the Methods. Information regarding the undisclosed four surveys is as follows: Reasons for controlled access: the MEXT does not allow users of the data to disclose it. Precise conditions of access (including contact details for access requests): it is necessary to get permission of the MEXT (Monbusho, shoto chuto kyoiku kyoku, kenko kyoiku shokuiku ka (Ministry of education, culture, sports, science and technology, Bureau of elementary and secondary education, Health and dietary education division). A time frame for response to requests: we submitted our FOIA request to the MEXT on 30 October 2020 and obtained the source data files from the MEXT on 24 December 2020 with their permission. Details of any restrictions imposed on data use via data use agreements: users should not disclose the data and cannot report analysis of the data so that readers can know the school closure status of a municipality.

## Source data

Source Data Fig. 1 Average outcomes and ATCs: main analysis.
Source Data Fig. 2 Treatment effects: controlling for covariates.
Source Data Fig. 3 Treatment effects: public health center fixed effects.
Source Data Fig. 4 Average outcomes and ATCs: conditioning on neighbors.
Source Data Fig. 5 Average outcomes and ATTs.
Source Data Extended Data Fig. 1 Sources of the daily number of newly confirmed cases: analyzed prefectures.
Source Data Extended Data Fig. 2 Definition of covariates.
Source Data Extended Data Fig. 3 Treatment effects: negative binomial regression.
Source Data Extended Data Fig. 4 Average outcomes and ATCs: inverse probability weighting.
Source Data Extended Data Fig. 5 Average outcomes and ATCs: a smaller set of covariates.

## Code availability

The replication code is deposited to Harvard Dataverse at 10.7910/DVN/N803UQ.
