# Supplementary material for: No causal effect of school closures in Japan on the spread of COVID-19 in spring 2020
Source: Nat Med. 2021 Oct 27;27(12):2111–9. doi: 10.1038/s41591-021-01571-8 (PMC8674136; doi:10.1038/s41591-021-01571-8)
Supplement: Supplementary file 1 — Supplementary Text, Figs. 1–5 and Tables 1–10 [file 41591_2021_1571_MOESM1_ESM.pdf]

nature portfolio

https://doi.org/10.1038/s41591-021-01571-8

Supplementary information

No causal effect of school closures in Japan on the spread of COVID-19 in spring 2020

In the format provided by the authors and unedited

Supplementary Information for “No causal effect of school closures in Japan on the spread of COVID-19 in spring 2020”

Kentaro Fukumoto* Charles T. McClean† Kuninori Nakagawa‡

October 20, 2021

*Professor, Department of Political Science, Gakushuin University. Mailing address: 1-5-1 Mejiro, Toshima-ku, Tokyo 171-8588, Japan. Email address: Kentaro.Fukumoto@gakushuin.ac.jp. Corresponding author.

†The majority of the work was completed when the author was a Postdoctoral Fellow in the Program on U.S.-Japan Relations at Harvard University. His current position is Toyota Visiting Professor, Center for Japanese Studies, University of Michigan, Weiser Hall, 500 Church Street, Suite 400, Ann Arbor, MI USA. Email address: cmcclean@umich.edu.

‡Associate Professor, Department of Economics, Shizuoka University. Mailing address: Ohya 836, Suruga-ku, Shizuoka, 422-8529, Japan. Email address: nakagawa.kuninori@shizuoka.ac.jp.

Data

Outcome variables

Table S1 displays the 20 non-target prefectures' URLs we refer to for the daily number of newly confirmed cases (accessed on August 12, 2020, though we updated the URLs (but not the data) on March 22, 2021). Note that The URLs where prefectures report COVID-19 information often change. For the these 20 prefectures, the aggregate outcome variables are available at the level of public health centers, which usually contain multiple municipalities, or even at the level of prefecture. Some prefectures aggregate towns and villages, not cities. Others aggregate all municipalities except their capital city (and a few cities that have their own public health centers). Hokkaido Prefecture aggregates outcome variables at the level of the General Development Bureau.

Table S1: Sources of the daily number of newly confirmed cases: not analyzed prefectures

Prefecture	Source
Hokkaido	`http://www.pref.hokkaido.lg.jp/hf/kth/kak/hasseijoukyou.htm`
Aomori	`https://www.pref.aomori.lg.jp/soshiki/kenko/hoken/index.html`
Miyagi	`https://www.pref.miyagi.jp/site/covid-19/02.html`
Akita	`https://www.pref.akita.lg.jp/pages/archive/47957`
Ibaraki	`https://www.pref.ibaraki.jp/1saigai/2019-ncov/index.html`
Gunma	`https://www.pref.gunma.jp/07/z87g_00016.html`
Kanagawa	`https://www.pref.kanagawa.jp/osirase/1369/`
Ishikawa	`https://www.pref.ishikawa.lg.jp/kansen/coronakennai.html`
Yamanashi	`https://www.pref.yamanashi.jp/koucho/coronavirus/info_coronavirus_preventio n.html`
Nagano	`https://www.pref.nagano.lg.jp/hoken-shippei/kenko/kenko/kansensho/joho/cor ona-doko.html`
Shizuoka	`https://www.pref.shizuoka.jp/kinkyu/covid-19-tyuumokujouhou.html`
Mie	`https://www.pref.mie.lg.jp/YAKUMUS/HP/m0068000066_00002.htm`
Kyoto	`http://www.pref.kyoto.jp/kentai/corona/hassei1-50.html`
Hyogo	`https://web.pref.hyogo.lg.jp/kk03/corona_hasseijyokyo.html`
Nara	`http://www.pref.nara.jp/55062.htm`
Wakayama	`https://www.pref.wakayama.lg.jp/prefg/041200/d00203387.html`
Kochi	`https://www.pref.kochi.lg.jp/soshiki/130401/2020022900049.html`
Fukuoka	`https://fukuoka.stopcovid19.jp/`
Kumamoto	`https://kumamoto.stopcovid19.jp/`
Okinawa	`https://www.pref.okinawa.lg.jp/site/hoken/chiikihoken/kekkaku/press/202002 14_covid19_pr1.html`

When the municipality where a case is confirmed is unknown (including quarantine at an airport, perhaps due to concerns about privacy protection), we do not count the case. When a patient who was confirmed in a municipality lived in another municipality, the data sources do not tell us the exact municipality where such a patient lived and thus we do not count these cases. Some prefectures report the date when a confirmed

case was announced, while others record the date when a case was confirmed.

We count cases from January 25, 2020. Before this date, we know of only two confirmed cases in Japan.[1] The first case was confirmed on January 15 in Kanagawa Prefecture, though the municipality is unknown.[2] The second case was confirmed on January 24 in Tokyo Prefecture, though the tourist's residence was in Wuhan, China.[3] Therefore, we do not count these two cases when we calculate the sum of all the outcome variables before the survey date.

Covariates

We take the log for variables that have no upper limit. We chose the covariates we did because they *could* affect both outcomes and treatments as confounders, and because data are available. Here are the reasons.

- Past treatment variables: once a municipality closed its schools, it may be more likely to do so later as well. And past school closures may have a longer-term effect to reduce the current outcomes.
- Sum of all the outcome variables before the survey date and the past seven days' outcome variables: the number of past cases is likely to affect the number of current cases and the likelihood that municipalities will close their schools.
- Prefecture dummies: a prefecture is in charge of NPIs other than school closures, and these NPIs were expected to reduce the outcome. Moreover, a prefecture may affect its municipalities' decisions about school closures as it does in other policy areas.
- Number of municipalities covered by the public health center that is in charge of a municipality: the more municipalities a public health center covers, the less patients it may be able to processes and the less cases may be counted. Expecting that, such municipalities may be more likely to close their schools.
- Five demographic variables (population, population density, the young, the old, and densely inhabited districts population): the more people live in a municipality, or the more densely people live in a municipality, or the more young/old people live in a municipality, the larger the outcome is likely to be. Expecting this possibility, these municipalities may be more likely to close their schools.

[1] Ministry of Health, Labour and Welfare (MHLW), "Shingata korona uirusu kansenshō nitsuite, ōpun dēta [Open data about COVID-19]," `https://www.mhlw.go.jp/stf/covid-19/open-data.html` (accessed on March 25, 2021).

[2] MHLW, "Shingata korona uirusu ni kanrenshita haien no kanja no hassei nitsuite (1 rei me) [On a patient of pneumonia related to SARS-CoV-2 (the first case)]," `https://www.mhlw.go.jp/stf/newpage_08906.html` (accessed on March 25, 2021).

[3] MHLW, "Shingata korona uirusu ni kanrenshita haien no kanja no hassei nitsuite (2 rei me) [On a patient of pneumonia related to SARS-CoV-2 (the second case)]," `https://www.mhlw.go.jp/stf/newpage_09079.html` (accessed on March 25, 2021).

- Seven commuting variables (in-migrants, out-migrants, commuters from other municipalities in the same prefecture, commuters from other prefectures, commuters to other municipalities in the same prefecture, commuters to other prefectures, and daytime population): the more people come and go, the more likely the outcome is to be larger and, expecting that, municipalities will tend to close their schools.

- Four geographic variables (inhabitable area size, the number of bordering municipalities, latitude, and longitude): the larger the inhabitable area or the more bordering municipalities a given municipality has, the larger the outcome values are likely to be. We match on latitude and longitude so that closer municipalities tend to be matched.

- Income and four variables on the municipal government's fiscal situation (financial solidity index, total revenue, local tax, and non-transferred revenue): wealthier municipalities may tend to be more urban and thus have larger outcome values and be more likely to close its schools.

- Four education variables (elementary school pupils, elementary school pupils per school, junior high school students, and junior high school students per school): if more children go to schools, the outcome may be likely to increase and, expecting that, municipalities will tend to close their schools.

- Four labor variables (labor force, unemployment, primary industry employment, and secondary industry employment): the more people work, the larger the outcome is likely to be. Expecting that, municipalities will tend to close their schools.

- Five medical variables (hospitals, medical clinics, beds of hospitals, beds of medical clinics, and physicians): the more medical institutions and workers there are, the more people will be tested and the more cases will be detected. Expecting that, municipalities will tend to close their schools.

- Three climatic variables (precipitation, daylight hours, and average temperature): worse climatic conditions (lesser precipitation, shorter daylight hours, and higher average temperature) may lead to higher outcomes and, expecting that, municipalities will tend to close their schools.

- Three mayoral variables (age, number of terms, days since last election): if past outcomes are large (which will lead to larger values of current outcomes), then more politically vulnerable mayors may tend to close the schools.

Government statistics

The definition of each original variable is at National Statistics Center, "Kōmoku teigi [Definition of items]" `https://www.e-stat.go.jp/koumoku/koumoku_teigi/A` (accessed on February 9, 2021). The outline of surveys is at National Statistics Center, "Tōkei Chōsatō no gaiyo [Outline of survey]" `https://www.e-stat.go.jp/koumoku/toukei_outline` (accessed on February 9, 2021). The definition of the financial solidity index (D2201) of the 23 special wards in Tokyo Prefecture is different from that for other municipalities (Tokyo Metropolitan Government, Bureau of General Affairs, Local Administration Division, "Yōgoshū [Glossary]" `https://www.soumu.metro.tokyo.lg.jp/05gyousei/dictionary.html`, accessed on February 8, 2021).

Matching

If we assume that (potential) outcomes are independent of treatment variables conditioned on the covariates (conditional unconfoundedness), the average of the matched treated municipalities' outcomes can identify the average of the counterfactual outcomes for the control municipalities if they were assigned treatment. Therefore, by subtracting the average of the control municipalities from that of the matched treated municipalities, we should be able to estimate the average treatment effect on the controlled (ATC) (50). Note that this identification strategy does not assume any modeling, while most empirical studies (implicitly) assume conditional unconfoundedness as well as some type of model (such as a linear model).

Main analysis

To achieve reproducibility, we take advantage of the `checkpoint` package (69) and specify the date of packages to use as May 25, 2021. It takes more than a dozen days in total to conduct all analyses. We regress the outcome variable on the treatment variable and no covariates using only the matched municipalities with matching weights (which take replacement into consideration). The coefficient of the treatment variable corresponds to the ATC. As for the treatment variable as of June 1, only two municipalities were treated so that proper inference on treatment effects is almost impossible. Therefore, we do not analyze the treatment variable.

Table S2 displays the number of matched treated and control municipalities by survey date. Note that the number of (matched) control municipalities is equal to that in Table 2. The number of matched treated municipalities is not larger than the number of (matched) control municipalities because some treated municipalities are matched to more than one control municipality.

Table S2: Numbers of matched treated and control municipalities by survey date

Survey date	Treated	Control
March 4	10	10
March 16	22	29
April 6	99	483
April 10	92	307
April 16	77	267
April 22	36	80
May 11	56	145

Figure S1 illustrates how matching improves balance in the covariates between the treated and control groups. One standard way to compare the differences across variables is the absolute standardized mean difference (ASMD), which is the absolute value of the difference in means, divided by the square root of the average of two group's variance (50). We use the `cobalt` package (70) for calculating ASMDs. In each panel, which corresponds to the treatment variable for a given survey date, the vertical and horizontal axes indicate the absolute standardized mean difference (ASMD) in a covariate between both groups before and after matching, respectively. (The bottom right panel deals with the ATT matching for the April 6 treatment variable.) Each circle represents one of the covariates, namely, zero to six past treatment variables, 25 or 26 prefecture dummy variables, and the other 49 covariates. When the ASMD of a covariate before or after matching is larger than two, we substitute a cross with a circle and place it at the value of two on the corresponding axis. If a circle is located below the 45 degree line, matching improves the balance in the corresponding covariate. Overall, for all survey dates, most of circles are below the 45 degree line, and matching improves balance in most of the covariates.

Figure S1: Improvement of covariate balance between the treated and control groups.

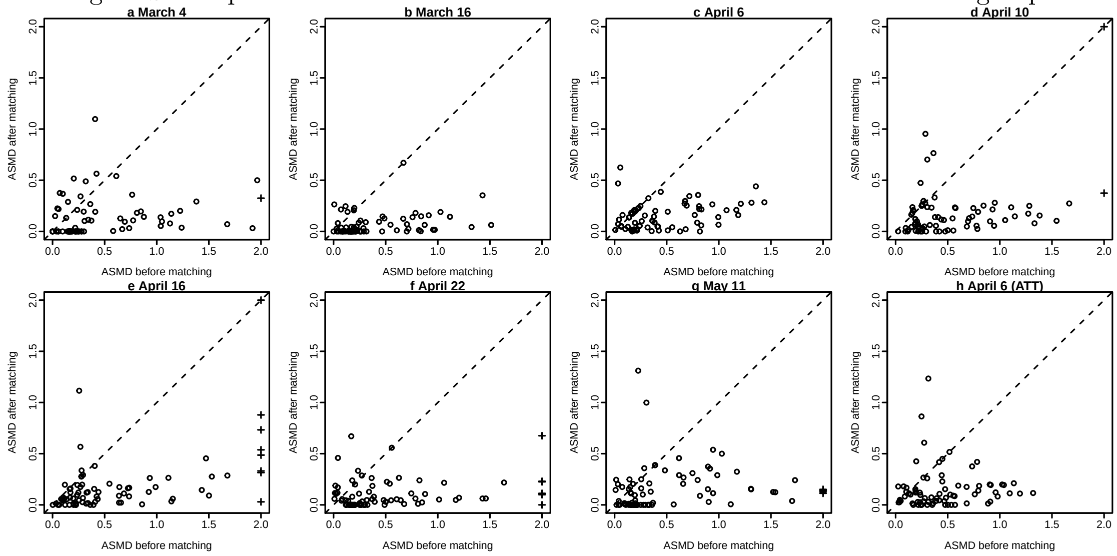

Note: The vertical and horizontal axes indicate the absolute standardized mean difference (ASMD) in a covariate between the treated and control groups before and after matching, respectively. Each circle or cross represents one of the covariates. A cross is used when its ASMD on an axis is larger than two.

To give readers a more concrete sense of what we mean, Table S3 shows the names of covariates in the descending order of the ASMD before matching, as an example, for the April 6 treatment variable. We match on 76 covariates (two past treatments (as of March 4 and 16), 25 prefecture dummies, and the other 49 variables). The first, second, and third columns display the ASMDs before matching (B), the ASMDs after matching (A), and the reduction ratio of the ASMDs (A/B). It seems that covariates related to urbanicity tend to be unbalanced between the two groups and thus could be confounders.

Table S3: Improvement of covariate balance between the treated and control groups for the treatment variable as of April 6

Variable name	ASMD		
	Before (B)	After (A)	Ratio (A/B)
Income	1.44	0.28	0.20
Densely inhabited districts population	1.36	0.44	0.33
Population density	1.31	0.28	0.21
Local tax	1.21	0.27	0.22
Commuters from other prefectures	1.19	0.16	0.13
Number of cases on April 3	1.17	0.21	0.18
Non transfered revenue	1.07	0.21	0.19
Number of cases on March 31	1.00	0.07	0.07
Livable area	0.99	0.14	0.14
Financial solidity index	0.94	0.26	0.28
1st industry employment	0.83	0.21	0.26
Cumulative number of cases	0.83	0.06	0.07
Number of cases on April 5	0.82	0.00	0.00
Old population ($\geq$ 65)	0.81	0.22	0.27
Aichi Prefecture dummy	0.81	0.25	0.30

(continued)

(continued from the previous page)

Variable name		ASMD	
	Before (B)	After (A)	Ratio (A/B)
Total revenue	0.80	0.36	0.44
Commuters to other prefectures	0.79	0.09	0.11
Elementary school pupils per school	0.77	0.16	0.21
Osaka Prefecture dummy	0.72	0.34	0.48
Junior high school students per school	0.69	0.25	0.37
Saitama Prefecture dummy	0.68	0.02	0.03
Population	0.68	0.30	0.44
Gifu Prefecture dummy	0.67	0.27	0.40
Daylight hours	0.63	0.00	0.00
Number of cases on April 4	0.55	0.04	0.07
Longitude	0.51	0.19	0.38
Commuters from other municipalities in the same prefecture	0.51	0.01	0.02
Fukui Prefecture dummy	0.44	0.39	0.87
Hospitals	0.42	0.01	0.02
Young population (<15)	0.42	0.05	0.12
Daytime population	0.41	0.05	0.11
Beds of medical clinics	0.39	0.20	0.51
Commuters to other municipalities in the same prefecture	0.38	0.09	0.23
In-migrants	0.38	0.15	0.38
Temperature	0.37	0.10	0.28
Precipitation	0.36	0.10	0.29
Junior high school students	0.36	0.00	0.01
Fukushima Prefecture dummy	0.32	0.32	1.00
Elementary school pupils	0.32	0.04	0.12
Chiba Prefecture dummy	0.29	0.16	0.54
Number of bordering municipalities	0.26	0.10	0.39
School closures as of March 16	0.25	0.25	1.00
2nd industry employment	0.24	0.06	0.23
Beds of hospitals	0.24	0.05	0.22
Mayor's age	0.23	0.23	0.99
Tokushima Prefecture dummy	0.23	0.23	1.00
Hiroshima Prefecture dummy	0.22	0.22	1.00
Niigata Prefecture dummy	0.21	0.01	0.04
Tochigi Prefecture dummy	0.21	0.02	0.09
Tottori Prefecture dummy	0.20	0.20	1.00
Shiga Prefecture dummy	0.20	0.20	1.00
Iwate Prefecture dummy	0.20	0.20	1.00
Shimane Prefecture dummy	0.20	0.20	1.00
Out-migrants	0.20	0.08	0.43
Miyazaki Prefecture dummy	0.19	0.01	0.05
Kagawa Prefecture dummy	0.19	0.19	1.00
Nagasaki Prefecture dummy	0.19	0.21	1.10
Latitude	0.19	0.02	0.11
Yamaguchi Prefecture dummy	0.18	0.01	0.06
Toyama Prefecture dummy	0.17	0.17	1.00
Oita Prefecture dummy	0.17	0.00	0.00
Medical clinics	0.17	0.02	0.10
Okayama Prefecture dummy	0.16	0.01	0.06
Number of mayor's terms	0.16	0.18	1.13
Days since the last mayoral election	0.14	0.14	0.97
School closures as of March 4	0.14	0.14	1.00
Saga Prefecture dummy	0.13	0.04	0.35
Number of municipalities covered by the public health center	0.10	0.02	0.23
Labor force	0.08	0.16	2.09
Number of cases on April 1	0.06	0.05	0.71
Number of cases on April 2	0.06	0.05	0.83
Yamagata Prefecture dummy	0.05	0.62	11.36
Unemployment	0.04	0.12	2.73
Physicians	0.03	0.07	2.15
Ehime Prefecture dummy	0.03	0.47	14.18
Number of cases on March 30	0.01	0.01	1.98

March 4

In February 2020, the number of COVID-19 cases began to increase in Japan. On the evening of February 27 (Thursday), Prime Minister Shinzo Abe abruptly "requested" that all schools close from March 2 (the following Monday) until their spring breaks, which were scheduled to take place from March 25 to April 5 for most schools.[4] In effect, this announcement meant schools would be closed for a month.[5] On the other hand, other NPIs were not imposed in March, at least officially (6). MEXT conducted the first survey of school closures on March 4. Thus, there is no any previous treatment variable to match on.

Of the 771 municipalities that have no missing values among our treatment, outcomes, and covariates for that survey date, only 10 (1.3%) rejected the prime minister's call and opened their schools. Since 9 of the 10 (90.0%) control municipalities also had open schools as of the next survey date (March 16), it is probable, but not certain, that they continued to open their schools between March 4 and 16. Similarly, 718 (94.3%) treated municipalities reported that their schools continued to be closed until March 16.[6] Therefore, note that the treatment variable represents whether schools are closed as of the survey date (i.e., March 4), not the dates of the outcome variables.

Since most municipalities are treated, the dotted black line in Fig. 1a is close to the national level of infection, which was modest. Nonetheless, both matched treated and control municipalities experienced no new COVID-19 cases during the study period (Fig. 1a). Therefore, the ATCs are zero (Fig. 1b).

March 16

Since spring break started on March 25 for most municipalities, as explained above, even control municipalities would effectively close their schools about a week following this survey date. It is worth noting that not all treated municipalities perfectly complied with the treatment. Among treated municipalities, 63.6% of them prepared spaces for students in their schools.[7] This number does not include special after-school care programs known as "gakudo" in Japan, where children study by themselves and play both inside and outside. Many of these programs remained in operation during the pandemic, including in municipalities with closed schools (in which case, children could stay there from morning to evening), but we lack more

[4] Specifically, the mode (48.6% of municipalities) of the beginning date of the spring break is March 25, and the mode (43.8% of municipalities) of the ending date of the spring break is April 5. Survey 1, the MEXT material for the treatments as of March 16, which we refer in Methods. Note that in Japan, an academic year ends in March.

[5] In fact, 80% of municipalities closed their schools until spring break began. Survey 2, the MEXT material for the treatments as of March 16, which we refer in Methods.

[6] Unlike control municipalities, we can know that these treated municipalities *continued* to close their schools from March 4 to March 16 according to Survey 2, the MEXT material for the treatments as of March 16, which we refer in Methods.

[7] Survey 2, the MEXT material for the treatments as of March 16, which we refer in Methods.

specific data as MEXT does not include these programs in its official survey since they are technically not part of schools.

We match on the past treatment variables as of March 4. Careful readers will find an uptick on April 1 (Fig. 1d). In Wadomari Town, Kagoshima Prefecture, which is in the control group, a case occurred among its 6,537 residents, which is equivalent to 15.3 cases per 100,000 residents. This single municipality contributes to 103.3% (= $(-15.3/29)/(-0.511)$) of the ATC (-0.511).[8] Therefore, this single outlier with a small population is mostly responsible for the large ATC on this day and thus the standard errors are also too large for us to reject the null hypotheses. Note that the mean population among all of the 785 municipalities in the 26 target prefectures is 76,830. The situation is similar to those for the ATCs of the April 16 treatment variable on the outcomes on April 23 and May 3, though we cannot disclose the identities of the two outlier municipalities due to the terms of use of the MEXT survey data.

April 6

Japan's academic year begins on April 1. As mentioned before, all schools had spring breaks for two weeks or so, mostly from March 25 to April 5, and other NPIs were not imposed in March, at least officially, either (6). Therefore, we do not have to care about the effects of past (i.e., March) NPIs including school closures when we study the effects of school closures as of April 6. (Such a situation does not hold for other survey dates except for March 4.) Anyway, recall that we match on the past two treatment variables (i.e., school status as of March 4 and 16).

For most schools, around the survey date of April 6, admission ceremonies were held for new students, and text books were distributed to all students. Probably for this reason, of the 739 municipalities with available data as of April 6, as many as 483 (65.4%) reported that their schools were open, which is the largest share among all of the survey dates and offers a desirable situation to estimate the ATCs in the sense that the number of treated municipalities and that of control municipalities are not lopsided as they are in March and after April 22 (Table 2). However, on April 7, the national government began to roll out a state of emergency, which gradually expanded from 7 prefectures to all 47 prefectures by April 16. Accordingly, prefectural governors started to issue NPIs. As a result, more and more municipalities closed their schools until 710 of the 790 (89.9%) municipalities had shut down their schools as of the April 22 survey. In fact, 247 of the 256 (96.5%) treated municipalities as of April 6 closed schools on all of the following three survey dates as well (April 10, 16, and 22), though only 79 of the 483 (16.4%) control municipalities opened their schools on all three dates.

In Fig. 1f, only on April 13, the 95% confidence interval of the ATC is slightly below zero.

[8] Recall that there were 29 control municipalities (Table 2).

April 10

On April 7, the national government issued a state of emergency for seven prefectures (Tokyo, Kanagawa, Saitama, Chiba, Osaka, Hyogo, Fukuoka). Accordingly, more municipalities closed their schools than on the previous survey date (Table 2). We match on the past three treatment variables (March 4, 16, and April 6).

April 16

On April 16, the state of emergency was extended to all 47 prefectures, while the national government additionally designated 13 prefectures as "prefectures on alert." Accordingly, more municipalities closed their schools than on the previous survey date (Table 2). Among treated municipalities, 59% of them prepared spaces in their schools (such as playing fields, gyms, classrooms, and libraries) for students (whose parents are, for instance, medical workers, essential workers, single parents, or disabled). As with the March 16 survey, this number does not include the "gakudo" after-school programs.[9] In this sense, these municipalities did not perfectly comply with the treatment. We match on the past four treatment variables (March 4, 16, April 6, and 10).

April 22

More municipalities closed their schools than on the previous survey date (Table 2). We find that 70 of the 80 (87.5%) control municipalities and 634 (89.3%) of 710 treated municipalities as of April 22 opened and closed their schools as of the next survey date (May 11), respectively. Thus, it is likely that the status of school closures did not change much between the two survey dates. We match on the past five treatment variables (March 4, 16, April 6, 10, and 16).

May 11

On May 4, the national government extended the end point of the state of emergency from May 6 to 31. Nonetheless, on May 14, 21, and 25, the national government lifted the state of emergency in 39, 3, and 5 prefectures, respectively. Thus, the state of emergency was over on May 25. Accordingly, just 2 of the 641 (0.3%) treated municipalities as of May 11 reported closed schools as of the next survey date (June 1). All of the control municipalities as of May 11 said that their schools were also open on June 1. We match on the past six treatment variables (March 4, 16, April 6, 10, 16, and 22). We can see that both outcome averages are almost equal to zero (Fig. 1m) and, therefore, the ATCs are also nearly zero (Fig. 1n).

[9] The MEXT material for the treatments as of April 16, which we refer in Methods.

Robustness checks

Negative binomial regression

In our analysis, the number of cases is equal to zero for 95.4% of our 79,989 municipality-day observations and less than 10 for 99.9% of our observations. Recall that our analysis covers all of the 847 municipalities in the 27 target prefectures from February 26 to June 1 (97 days). Note also that the number of cases is not available for 62 municipalities in Tokyo Prefecture from February 26 to March 31 (35 days). Thus, the total number of units is $847 \times 97 - 62 \times 35 = 79,989$. Figure S2 presents the histogram of the number of cases.

Figure S2: Histogram of the number of cases.

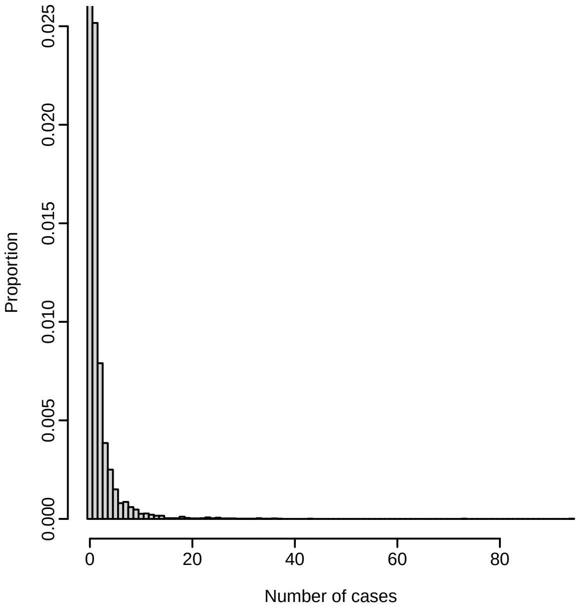

Note: The unit of analysis is a municipality and a day ($N = 79,989$). We analyze 847 municipalities in the 27 target prefectures from February 26 to June 1 (97 days). The proportion of units with zero cases is 0.954 and truncated.

Public health center fixed effects

Figure S3 displays the distribution of the number of municipalities per public health center. It turns out that in the 27 target prefectures, 66.9% of the 263 public health centers cover multiple municipalities, which occupy 89.7% of the 847 municipalities.

Nonetheless, not all public health centers with multiple municipalities contribute to the identification of treatment effects. Only "mixed" public health centers that have responsibility for both treated and control municipalities do so. Table S4 counts the overall total and number of mixed public health centers in the

Figure S3: Histogram of the number of municipalities per public health center.

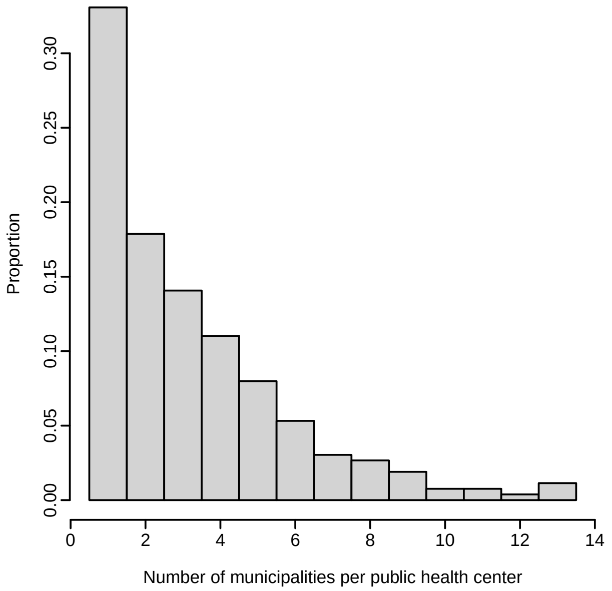

Note: The unit of analysis is a municipality ($N = 847$). We analyze 847 municipalities and 263 public health centers in the 27 target prefectures.

first and second columns, respectively, where each row corresponds to each survey date. If all municipalities in a public health center have missing values of any variable we use, the public health center is not count. Therefore, the overall total of mixed public health centers is less than 263.

Table S4: Number of public health centers

Survey date	All	Mixed
March 4	231	4
March 16	230	14
April 6	229	28
April 10	260	18
April 16	258	23
April 22	258	12
May 11	257	16

Note: The unit of analysis is a public health center.

Table S5 presents the numbers of municipalities by public health center status and treatment status. Each row corresponds to each survey date. The first to third columns indicate the number of treated, control, and all municipalities covered by mixed public health centers we analyze, respectively. The fourth to sixth columns correspond to the case of non-mixed public health centers.[10] Though we analyze all of these

[10]The sum of municipalities covered by mixed and non-mixed public health centers (e.g., the first and fourth columns) is equal to the corresponding number (e.g., the first column) in Table 2.

municipalities, in effect, the public health center fixed effects model exploits variation in outcomes among only municipalities covered by mixed public health centers.

Table S5: Number of municipalities by public health center status and treatment status

	Mixed health center			Non-mixed health center		
Survey date	Treated	Control	All	Treated	Control	All
March 4	18	7	25	743	3	746
March 16	51	20	71	667	9	676
April 6	62	77	139	194	406	600
April 10	24	65	89	467	242	709
April 16	35	53	88	488	214	702
April 22	30	24	54	680	56	736
May 11	43	25	68	598	120	718

Note: The unit of analysis is a municipality.

Inverse probability weighting

We denote the propensity score, the probability that a municipality is treated conditioned on covariates, as p. Assuming conditional unconfoundedness, we can identify the average of if-treated outcomes of control municipalities by the weighted average outcome of the treated municipalities where the weight is $(1-p)/p$. By subtracting the average outcome of the control municipalities from the weighted average of the treated municipalities, we can estimate the ATC (67).

On March 4 (Extended Data Fig. 2a) and April 10 (Extended Data Fig. 2g), all treated municipalities are equally weighted so that the weighted and un-weighted average outcomes of the treated municipalities overlap completely (the dotted black line exactly overlaps the black solid line and is thus invisible). That is, inverse probability weighting does not work well for these two treatment variables. This is a reason why we prefer matching to inverse probability weighting with this particular data. We did not correct for multiple comparisons.

Conditioning on neighbors

At first, we retain all of 1,741 municipalities in all of 47 prefectures. We drop off a municipality if any of its neighbor municipalities has a missing value of the treatment variable or if the municipality has no neighbor municipalities (e.g., an island). Then, we apply the same selection procedure to the remaining municipalities as the main analysis. The fourth column of Table S6 represents the number of the selected municipalities, which is less than the third column of Table 2. We divide the selected municipalities into three classes: all neighbors are treated ("treated", first column of Table S6), no neighbors are treated ("control", second

column), and some neighbors are treated but others are not ("mixed", third column).[11] For all of the survey dates except April 6, the most frequent pattern was the situation where a municipality was surrounded by only treated municipalities. Figure S4 illustrates the distribution of the percentage of treated neighbors across 678 municipalities on April 6. The situation where all neighbors are treated was the second most frequent.

Table S6: Number of municipalities by treatment status of neighbors

	Neighboring municipalities			
Survey date	Treated	Control	Mixed	All
March 4	711	0	29	740
March 16	596	2	72	670
April 6	130	338	210	678
April 10	379	178	185	742
April 16	399	133	156	688
April 22	604	26	92	722
May 11	504	66	139	709

Note: The unit of analysis is a municipality. We drop off a municipality if any of its neighbor municipalities has a missing value of the treatment variable or if the municipality has no neighbor municipalities. We consider neighbors outside the 26 or 27 target prefectures as well.

Figure S4: Histogram of the percentage of treated neighbors on April 6.

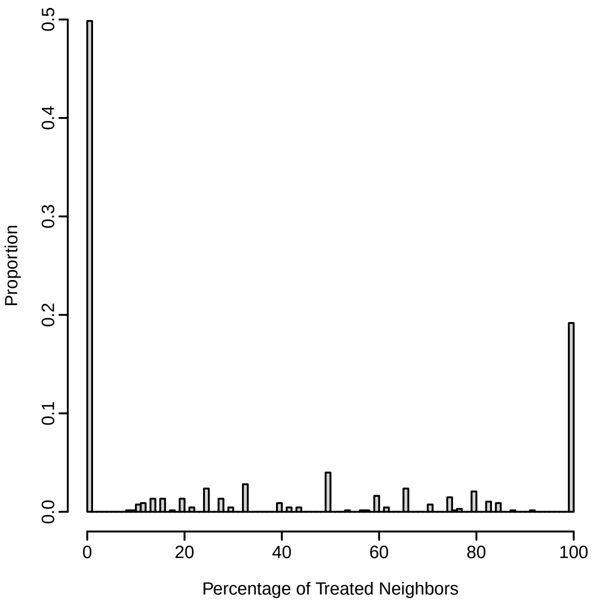

Note: The unit of analysis is a municipality ($N = 678$). We drop off a municipality if any of its neighbor municipalities has a missing value of the treatment variable or if the municipality has no neighbor municipalities. We consider neighbors outside the 26 or 27 target prefectures as well.

[11]Neighbors may not belong to the 26 or 27 target prefectures.

Table S7 summarizes the numbers of treated and control municipalities surrounded by treated municipalities. Note the sum of the two columns in Table S7 is equal to the first column of Table S6. Even though we conduct matching with replacement, it turns out that no treated municipality is matched to more than one control municipality.

Table S7: Numbers of treated and control municipalities surrounded by treated municipalities

Survey date	Treated	Control
March 4	709	2
March 16	590	6
April 6	125	5
April 10	378	1
April 16	397	2
April 22	600	4
May 11	500	4

Note: The unit of analysis is a municipality.

A smaller set of covariates

We match on a smaller set of covariates that excludes the following 25 covariates from our main analysis: one of the five demographic variables (the young); six of the seven commuting variables (in-migrants, out-migrants, commuters from other municipalities in the same prefecture, commuters from other prefectures, commuters to other municipalities in the same prefecture, and commuters to other prefectures); four geographic variables (inhabitable area size, the number of bordering municipalities, latitude, and longitude); two of the four variables on the municipal government's fiscal situation (total revenue and non-transferred revenue); four labor variables (labor force, unemployment, primary industry employment, and secondary industry employment); two of the five medical variables (beds of hospitals and beds of medical clinics); three climatic variables (precipitation, daylight hours, and average temperature); and three mayoral variables (age, number of terms, days since last election).

External validity

Our findings are based on municipalities in the 26 or 27 target prefectures that do not have missing values for any variable. Table S8 displays the number of municipalities analyzed by this paper (first column, which corresponds to the third column of Table 2), missing municipalities inside the target prefectures (second column), and municipalities outside the target prefectures (third column). Readers may wonder if our results can be extended to the last two cases. It is challenging to answer this question; these municipalities

are excluded from our study exactly because they have missing values and cannot be used for estimating the effect of school closures on the spread of COVID-19.

Table S8: Number of municipalities by analysis status

Survey date	Inside the target Analyzed	Missing	Outside the target
March 4	771	14	956
March 16	747	38	956
April 6	739	46	956
April 10	798	49	894
April 16	790	57	894
April 22	790	57	894
May 11	786	61	894

With that being said, we present some suggestive information here. If the averages of covariates and outcomes are significantly different between the analyzed municipalities and the missing municipalities inside the target prefectures (or municipalities outside the target prefectures), it is unlikely that we can extend our findings to those un-analyzed municipalities. Otherwise, we may be able to conjecture that our results hold for the un-analyzed municipalities as well.

First, we compare the averages of covariates between the analyzed municipalities and the missing municipalities inside the target prefectures. We apply t-tests to the 49 continuous covariates and Fisher exact tests to the binary covariates (past treatment variables and the prefecture dummies). If a municipality has a missing value for a covariate, we exclude the municipality from the test of the covariate (pairwise deletion). Table S9 summarizes the results. The first column reports the number of all covariates. The second column shows the number of covariates that are significantly different between the two groups at the 5% significance level. The third column indicates the percentage returned by dividing the second column by the first column. Overall, the averages of almost half of covariates are not similar between the two groups, and thus we cannot be optimistic about the generalizability of our results to these municipalities.

Table S9: Number of covariates: missing municipalities

Survey date	All	Different	%
March 4	74	29	39.2
March 16	75	37	49.3
April 6	76	32	42.1
April 10	78	36	46.2
April 16	79	36	45.6
April 22	80	33	41.2
May 11	81	42	51.9

We next compare the averages of *outcomes* between the two groups. In Figure S5a, the black and red lines represent the averages of outcomes in the analyzed municipalities and the missing municipalities inside the target prefectures, respectively. The eight vertical turquoise lines correspond to the survey dates. For the dates between surveys, we show the averages of outcomes using the treatment status as identified by the earlier survey. For instance, the black line between the second turquoise line (March 16) and the third turquoise line (April 6) represents the averages of outcomes in the analyzed municipalities for the second treatment variable (March 16). In Figure S5b, the thick black line demonstrates the differences in the two outcome averages, where the shaded grey area represents the 95% confidence intervals. The differences are never significantly different from zero. This figure is thus more favorable toward the generalizability of our results.

Figure S5: Comparison of outcomes between analyzed and missing municipalities.

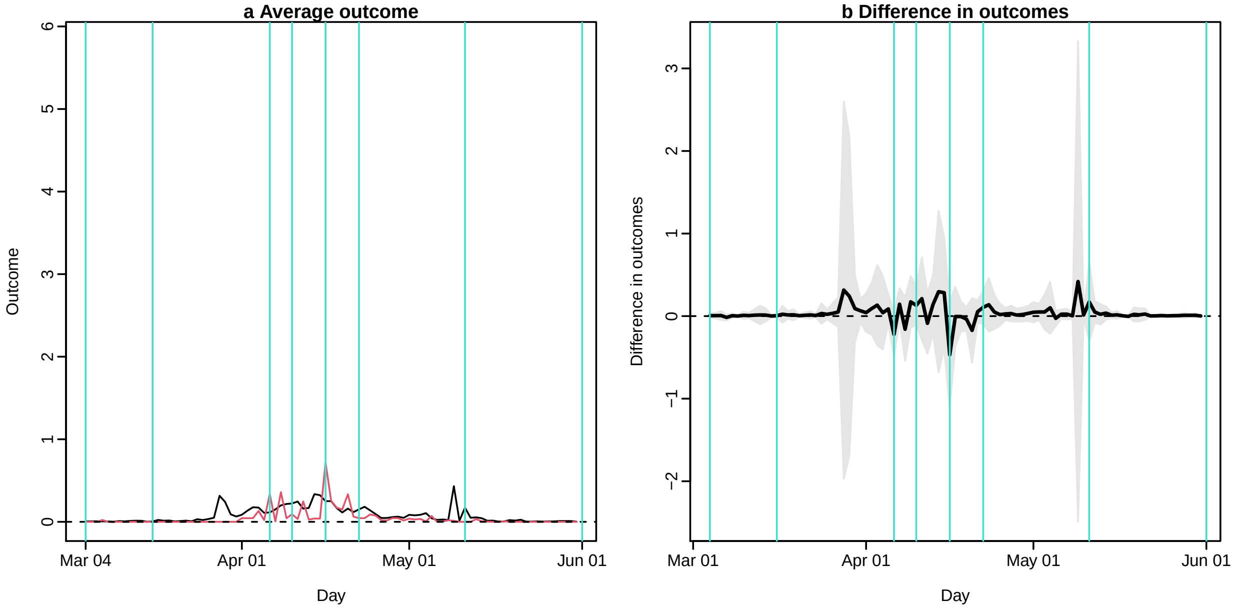

Note: The eight vertical turquoise lines correspond to the survey dates. **a**, The black and red lines represent the averages of outcomes in the analyzed municipalities and the missing municipalities inside the target prefectures, respectively. **b**, The thick black line demonstrates the differences in the two outcome averages, where the shaded grey area represents the 95% confidence intervals.

Second, we compare the averages of covariates between the analyzed municipalities and the municipalities *outside* the target prefectures. We do not examine prefecture dummies and the past seven days' outcome variables. Table S10 reports the results like Table S9. Since the averages of more than 70% of covariates are different between the two groups, we are pessimistic about the generalizability of our results to municipalities in these prefectures. We are unable to compare outcomes between the two groups because the outcome values are unavailable in many municipalities outside the target prefectures.

Table S10: Number of covariates: outside prefectures

Survey date	All	Different	%
March 4	42	30	71.4
March 16	43	31	72.1
April 6	44	32	72.7
April 10	45	32	71.1
April 16	46	33	71.7
April 22	47	33	70.2
May 11	48	34	70.8

To conclude, we are mostly not confident that our findings can be extended outside the analyzed municipalities. However, there is one caveat: even if the average of a covariate is different between two groups, matching would be able to adjust the difference if the necessary data becomes available. Thus, this section is not optimistic about the generalizability of our findings given the current data availability but does not deny it.

References

[69] de Vries, A. & Microsoft. checkpoint: Install packages from snapshots on the checkpoint server for reproducibility. R package version 0.4.10. `https://CRAN.R-project.org/package=checkpoint` (2020).

[70] Noah, G. cobalt: Covariate balance tables and plots. R package version 4.3.1. `https://CRAN.R-project.org/package=cobalt` (2021).
