# Supplementary material for: No causal effect of school closures in Japan on the spread of COVID-19 in spring 2020
Source: Nat Med. 2021 Oct 27;27(12):2111–9. doi: 10.1038/s41591-021-01571-8 (PMC8674136; doi:10.1038/s41591-021-01571-8)
Supplement: Supplementary file 2 — Reporting Summary [file 41591_2021_1571_MOESM2_ESM.pdf]

nature portfolio

Corresponding author(s): Kentaro Fukumoto

Last updated by author(s): Sep 30, 2021

Reporting Summary

Nature Portfolio wishes to improve the reproducibility of the work that we publish. This form provides structure for consistency and transparency in reporting. For further information on Nature Portfolio policies, see our Editorial Policies and the Editorial Policy Checklist.

Statistics

For all statistical analyses, confirm that the following items are present in the figure legend, table legend, main text, or Methods section.

n/a	Confirmed	
☐	☒	The exact sample size (*n*) for each experimental group/condition, given as a discrete number and unit of measurement
☐	☒	A statement on whether measurements were taken from distinct samples or whether the same sample was measured repeatedly
☒	☐	The statistical test(s) used AND whether they are one- or two-sided<br>*Only common tests should be described solely by name; describe more complex techniques in the Methods section.*
☐	☒	A description of all covariates tested
☐	☒	A description of any assumptions or corrections, such as tests of normality and adjustment for multiple comparisons
☐	☒	A full description of the statistical parameters including central tendency (e.g. means) or other basic estimates (e.g. regression coefficient) AND variation (e.g. standard deviation) or associated estimates of uncertainty (e.g. confidence intervals)
☒	☐	For null hypothesis testing, the test statistic (e.g. *F*, *t*, *r*) with confidence intervals, effect sizes, degrees of freedom and *P* value noted<br>*Give P values as exact values whenever suitable.*
☒	☐	For Bayesian analysis, information on the choice of priors and Markov chain Monte Carlo settings
☒	☐	For hierarchical and complex designs, identification of the appropriate level for tests and full reporting of outcomes
☒	☐	Estimates of effect sizes (e.g. Cohen's *d*, Pearson's *r*), indicating how they were calculated

Our web collection on statistics for biologists contains articles on many of the points above.

Software and code

Policy information about availability of computer code

Data collection	No software was used for data collection purposes.
Data analysis	R: A Language and Environment for Statistical Computing (Version 4.0.3). The R packages we use are tidyverse (version 1.3.1), checkpoint (version 0.4.10), MatchIt (version 4.1.0), Matching (version 4.9-9), rgenoud (version 5.8-3.0), lmtest (version 0.9-38), sandwich (version 3.0-1), lfe (version 2.8-3), MASS (version 7.3-53), CBPS (version 0.22), WeightIt (version 0.11.0), survey (version 4.0), and cobalt (version 4.3.1). The replication code is deposited to Harvard Dataverse, https://doi.org/10.7910/DVN/N803UQ.

For manuscripts utilizing custom algorithms or software that are central to the research but not yet described in published literature, software must be made available to editors and reviewers. We strongly encourage code deposition in a community repository (e.g. GitHub). See the Nature Portfolio guidelines for submitting code & software for further information.

Data

Policy information about availability of data

All manuscripts must include a data availability statement. This statement should provide the following information, where applicable:

- Accession codes, unique identifiers, or web links for publicly available datasets
- A description of any restrictions on data availability
- For clinical datasets or third party data, please ensure that the statement adheres to our policy

All data except the four surveys (as of April 16, 22, May 11, and June 1, 2020) from the Japanese Ministry of Education, Culture, Sports, Science and Technology (MEXT) is deposited to Harvard Dataverse as Fukumoto, Kentaro; Charles T. McClean; Kuninori Nakagawa, 2021, “Replication Data for: No causal effect of school closures in Japan on the spread of COVID-19 in spring 2020,” at https://doi.org/10.7910/DVN/N803UQ. All the data sources are detailed in Methods section, Data subsection. Information of the non-disclosed four surveys is as follows.

Reasons for controlled access: MEXT does not allow users of the data to disclose it.
Precise conditions of access (including contact details for access requests): it is necessary to get permission of MEXT (Monbusho, shoto chuto kyoiku kyoku, kenko kyoiku shokuiku ka [Ministry of education, culture, sports, science and technology, Bureau of Elementary and secondary education bureau, Health and dietary education division] (address: 3-2-2 Kasumigaseki, Chiyoda-ku, Tokyo-to 100-8959 Japan).
A timeframe for response to requests: We submitted our FOIA request to MEXT on October 30, 2020 and obtained the source data files from MEXT on December 24, 2020 with their permission.
Details of any restrictions imposed on data use via data use agreements: Users should not disclose the data and cannot report analysis of the data so that readers can know the school closure status of a municipality.

Field-specific reporting

Please select the one below that is the best fit for your research. If you are not sure, read the appropriate sections before making your selection.

☐ Life sciences ☒ Behavioural & social sciences ☐ Ecological, evolutionary & environmental sciences

For a reference copy of the document with all sections, see nature.com/documents/nr-reporting-summary-flat.pdf

Behavioural & social sciences study design

All studies must disclose on these points even when the disclosure is negative.

Study description	For every set of survey date and outcome date, we implement a cross-section analysis across 847 municipalities where we perform matching and regress the outcome variable on the treatment variable using only the matched municipalities. The survey dates are March 4, 16, April 6, 10, 16, 22, and May 11. The outcome dates range from February 26 to June 1, 2020.
Research sample	847 municipalities in Japan. They are not necessarily representative. They are chosen because the number of COVID-19 cases is available.
Sampling strategy	847 municipalities in Japan are chosen because data on the daily number of COVID-19 cases is available.
Data collection	We download the data from various websites. Those who maintain the data recorded the data.
Timing	From February 26 to June 1, 2020.
Data exclusions	No data were excluded from the analyses.
Non-participation	This item is not applicable because our study is observational.
Randomization	Municipalities were not allocated into experimental groups.

Reporting for specific materials, systems and methods

We require information from authors about some types of materials, experimental systems and methods used in many studies. Here, indicate whether each material, system or method listed is relevant to your study. If you are not sure if a list item applies to your research, read the appropriate section before selecting a response.

Materials & experimental systems

n/a	Involved in the study	
☒	☐	Antibodies
☒	☐	Eukaryotic cell lines
☒	☐	Palaeontology and archaeology
☒	☐	Animals and other organisms
☒	☐	Human research participants
☒	☐	Clinical data
☒	☐	Dual use research of concern

Methods

n/a	Involved in the study	
☒	☐	ChIP-seq
☒	☐	Flow cytometry
☒	☐	MRI-based neuroimaging
